# Exogenous Methyl Jasmonate and Salicylic Acid Induce Subspecies-Specific Patterns of Glucosinolate Accumulation and Gene Expression in *Brassica oleracea* L.

**DOI:** 10.3390/molecules21101417

**Published:** 2016-10-24

**Authors:** Go-Eun Yi, Arif Hasan Khan Robin, Kiwoung Yang, Jong-In Park, Byung Ho Hwang, Ill-Sup Nou

**Affiliations:** 1 Department of Horticulture, Sunchon National University, Suncheon-si 540-950, Korea; yeege91@hanmail.net (G.-E.Y.); gpb21bau@gmail.com (A.H.K.R.); ykw7685@naver.com (K.Y.); jipark@sunchon.ac.kr (J.-I.P.); 2 R & D Center for Crop Breeding, Asia Seed Ltd., Icheon-si, Gyeonggi-do 467-906, Korea; bhhwang0915@hanmail.net

**Keywords:** glucosinolates, biosynthetic genes, expression analysis, *Brassica oleracea*, subspecies, methyl jasmonate, salicylic acid

## Abstract

Glucosinolates have anti-carcinogenic properties. In the recent decades, the genetics of glucosinolate biosynthesis has been widely studied, however, the expression of specific genes involved in glucosinolate biosynthesis under exogenous phytohormone treatment has not been explored at the subspecies level in *Brassica oleracea*. Such data are vital for strategies aimed at selective exploitation of glucosinolate profiles. This study quantified the expression of 38 glucosinolate biosynthesis-related genes in three *B. oleracea* subspecies, namely cabbage, broccoli and kale, and catalogued associations between gene expression and increased contents of individual glucosinolates under methyl jasmonate (MeJA) and salicylic acid (SA) treatments. Glucosinolate accumulation and gene expression in response to phytohormone elicitation was subspecies specific. For instance, cabbage leaves showed enhanced accumulation of the aliphatic glucoiberin, progoitrin, sinigrin and indolic neoglucobrassicin under both MeJA and SA treatment. MeJA treatment induced strikingly higher accumulation of glucobrassicin (GBS) in cabbage and kale and of neoglucobrassicin (NGBS) in broccoli compared to controls. Notably higher expression of *ST5a* (Bol026200), *CYP81F1* (Bol028913, Bol028914) and *CYP81F4* genes was associated with significantly higher GBS accumulation under MeJA treatment compared to controls in all three subspecies. *CYP81F4* genes, trans-activated by *MYB34* genes, were expressed at remarkably high levels in all three subspecies under MeJA treatment, which also induced in higher indolic NGBS accumulation in all three subspecies. Remarkably higher expression of *MYB28* (Bol036286), *ST5b*, *ST5c*, *AOP2*, *FMOGS-OX5* (Bol031350) and *GSL-OH* (Bol033373) was associated with much higher contents of aliphatic glucosinolates in kale leaves compared to the other two subspecies. The genes expressed highly could be utilized in strategies to selectively increase glucosinolate compounds in *B. oleracea* subspecies. These results promote efforts to develop genotypes of *B. oleracea* and other species with enhanced levels of desired glucosinolates.

## 1. Introduction

Glucosinolates are sulfur-rich secondary metabolites derived from amino acids and sugars that are biosynthesized in plant tissues. These molecules are widely produced in all oilseed and vegetable species of the order Brassicales, including *Brassica oleracea* [1]. In fact, the hydrolysis of glucosinolates imparts characteristic flavors to *Brassica* vegetables [2,3]. Myrosinase enzymes play the key role in hydrolysis of glucosinolates into bioactive and anti-carcinogenic products such as thiocyanates, isothiocyanates, nitrile and erucin [4,5,6]. Glucosinolate compounds help prevent cancer cell production in animal tissues by controlling the cell cycle and accelerating apoptosis [7]. Sulforaphane (an isothiocyanate) [8,9] and indole-3-carbinol (a product of isothiocyanate) [10] are strongly anti-carcinogenic, whereas phenethyl isothiocyanate plays an inhibitory role in the conversion of potential carcinogens from their native forms into carcinogenic forms [11,12]. The products of glucosinolate hydrolysis can also induce important detoxifying enzymes, for example, glutathione *S*-transferase and quinone reductase (QR) [8,13,14]. QR catalyzes the conversion of toxic quinones into stable and non-toxic hydroquinones, reducing oxidative cycling [15], and the activation of QR has often been used as a biomarker for cancer prevention. The products of glucosinolate hydrolysis also up-regulate other health-promoting bioactivities including anti-inflammatory activity in *B. oleracea* [16,17]. However, not all glucosinolate compounds play equivalent roles in human health and plant defense. For example, indolic glucobrassicin has the greatest antioxidative effect compared to other glucosinolates [18,19]. Moreover, an anti-nutritional effect, e.g., goitrogenic effect (anti-thyroid activity) of progoitrin in ruminant animals is also reported [20].

Elucidating the responses governing glucosinolate biosynthesis and accumulation with regard to exogenous factors is important in designing a strategy to produce *Brassica* vegetable varieties enriched in glucosinolates beneficial for human health and plant protection. A number of biotic and abiotic stresses increase the biosynthesis and accumulation of different types of glucosinolates in *Brassica* species. Exogenous application of jasmonic acid (JA) or salicylic acid (SA) is often used to mimic biotic stress. In notable number of studies, JA and SA applications have been shown to increase accumulation of beneficial biomolecules, including glucosinolates [21,22,23,24,25,26,27,28,29,30,31,32,33]. Methyl jasmonate (MeJA) can be utilized in fields for *Brassica* vegetable production to enhance human health-promoting glucosinolates and the market value of the products [25]. Experimental evidence suggests that MeJA-induced glucosinolates enhance QR activity and, thus, play anti-carcinogenic roles [27]. Exogenous MeJA has been reported to enhance particular indolic glucosinolates; for example, neoglucobrassicin significantly accumulated in the leaves of *Brassica* crops such as pak choi [34], cabbage [35], oilseed rape [36], broccoli [37], Chinese kale [38], oilseed mustard [39] and turnip [40]. By contrast, exogenous SA treatment has been reported to stimulate the biosynthesis and accumulation of all three types of glucosinolates: aromatic, indole, and aliphatic glucosinolates in *Brassica* crops [21,24,38,40]. Pre-treatment with MeJA four days before harvest significantly improves the contents of desirable glucosinolates in kale, broccoli and cauliflower without decreasing postharvest quality [25,26,27,28]. Accordingly, in our present study, we decided to apply MeJA and SA treatment four days before sampling to measure levels of aliphatic and indolic glucosinolates as well as the expression of genes involved in accumulation of glucosinolates.

In the *Brassicaceae* family, glucosinolate biosynthesis is accomplished through a specially featured three steps process, namely: (i) elongation of the amino acid side chain; (ii) core structure formation; and (iii) secondary modifications of side chains. *MYB*-transcription factor related genes that trans-activate the functions of several genes are vital for side chain elongation and core-structure formation [41]. A notable number of different gene loci are involved in secondary modifications of desulfo-glucosinolates to produce characteristically different glucosinolates, for example: *ST5*, *GS-OX*, *GS-AOP*, *GS-OH* are involved in aliphatic glucosinolate biosynthesis and *CYP81*, *IGM* are involved in indolic glucosinolate biosynthesis. It is therefore important to investigate which particular genes are involved in enhancing glucosinolates under exogenous phytohormone elicitation.

*B. oleracea* is an important *Brassica* vegetable species that includes a number of commercially valuable subspecies, such as cabbage, cauliflower, broccoli, kale, kohlrabi, and Brussels sprouts, among others. In *B. oleracea*, about 105 glucosinolate metabolism-related genes have been identified, including 22 catabolism-related genes [42]. Reverse transcription-PCR (RT-PCR)-based expression profiling of 84 genes associated with glucosinolate transcription, biosynthesis and breakdown recently revealed that not all of the genes are expressed in the edible organs of various *B. oleracea* subspecies [43]. However, expression patterns of glucosinolate biosynthetic genes after exposure to phytohormone elicitors has not been extensively explored in *B. oleracea*, although this information is needed for selective enhancement of healthful glucosinolate compounds. In this study, we have selected three economically important *B. oleracea* subspecies in Korea: cabbage, broccoli and kale.

Here, we aimed to relate the expression of glucosinolate biosynthesis genes in three selected *B. oleracea* subspecies to their glucosinolate contents with or without MeJA and SA treatment. Identifying the genes that underlie the higher glucosinolate biosynthesis mediated by exogenous MeJA and SA at the subspecies level will open a window to generate novel commercial cultivars of *B. oleracea* with enhanced contents of desired glucosinolates.

## 2. Results

### 2.1. Subspecies-Specific Effects of Exogenous MeJA or SA on Glucosinolate Biosynthesis Gene Expression

**Figure 1:**
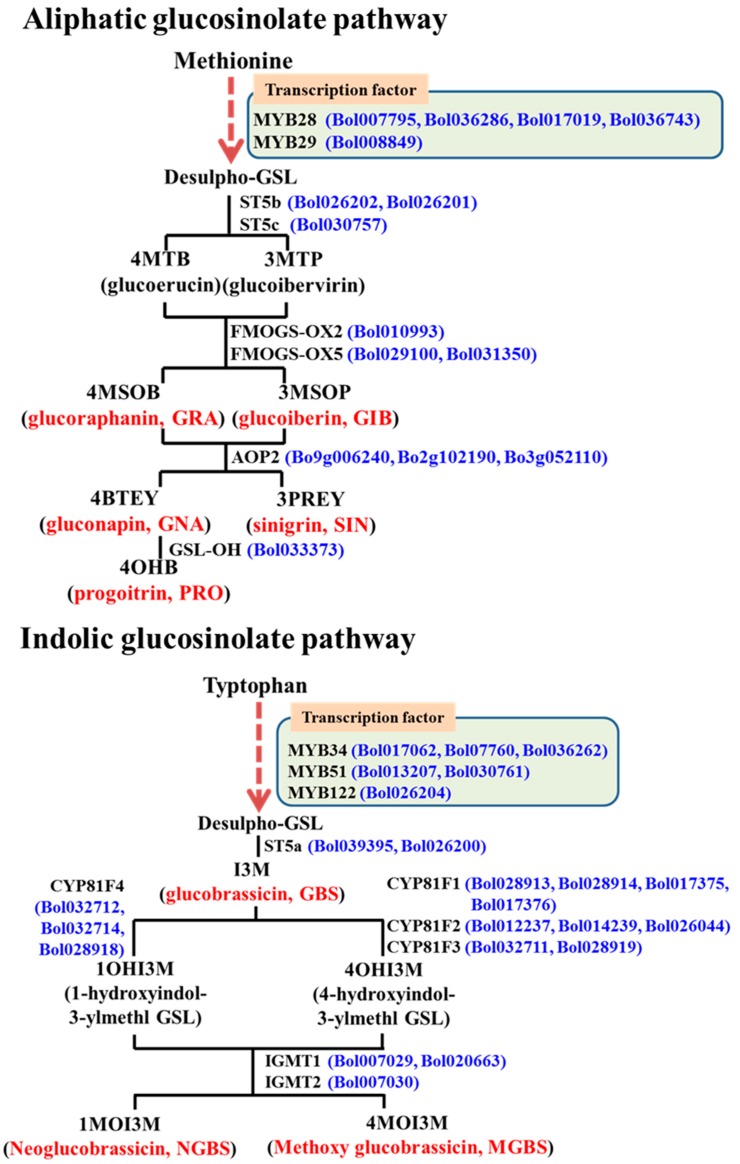
The 38 genes (blue letters) analyzed in this work, with their postions in aliphatic and indolic glucosinolate (GSL, red letters) biosynthesis pathways. A total of 15 and 23 genes were selected from aliphatic and indolic glucosinolate biosynthesis pathways, respectively. Chemical structures of the glucosinolate compounds and intermediates are presented in Figure S2.

**Figure 2:**
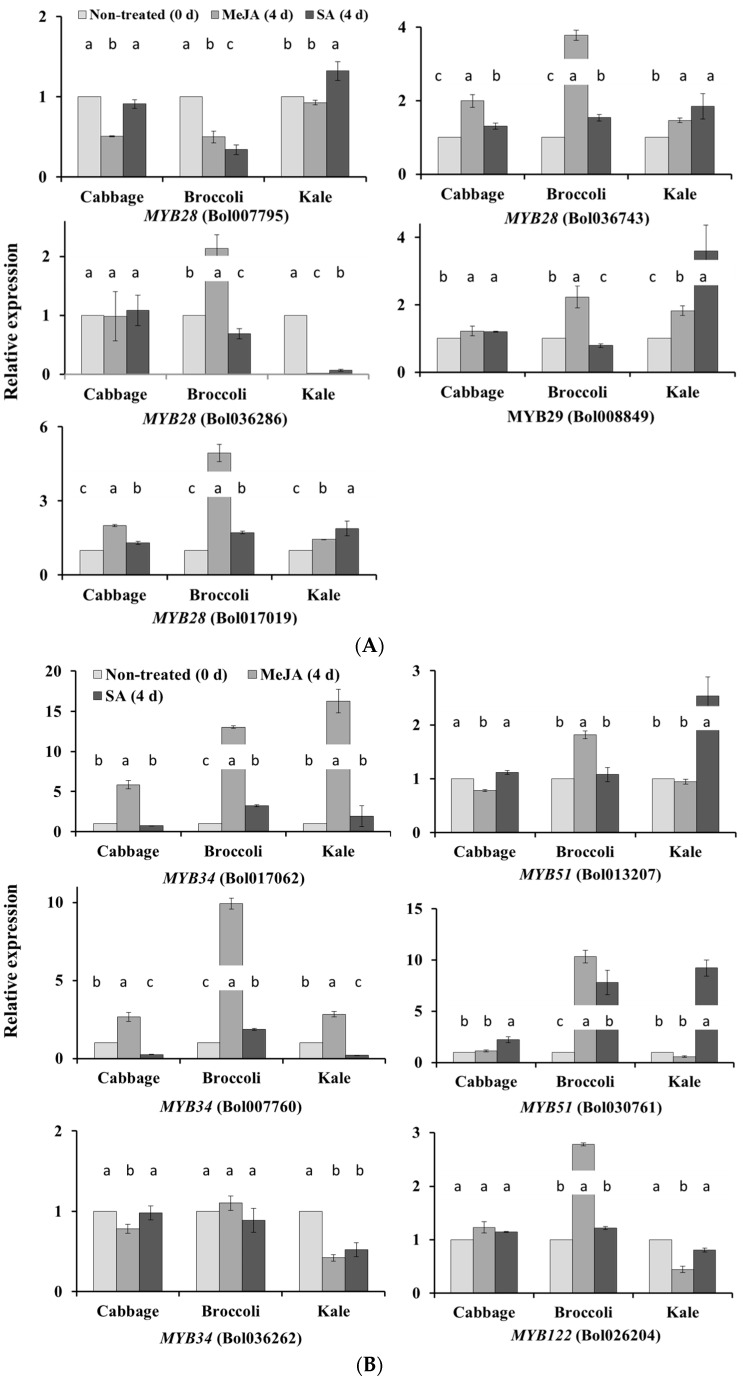

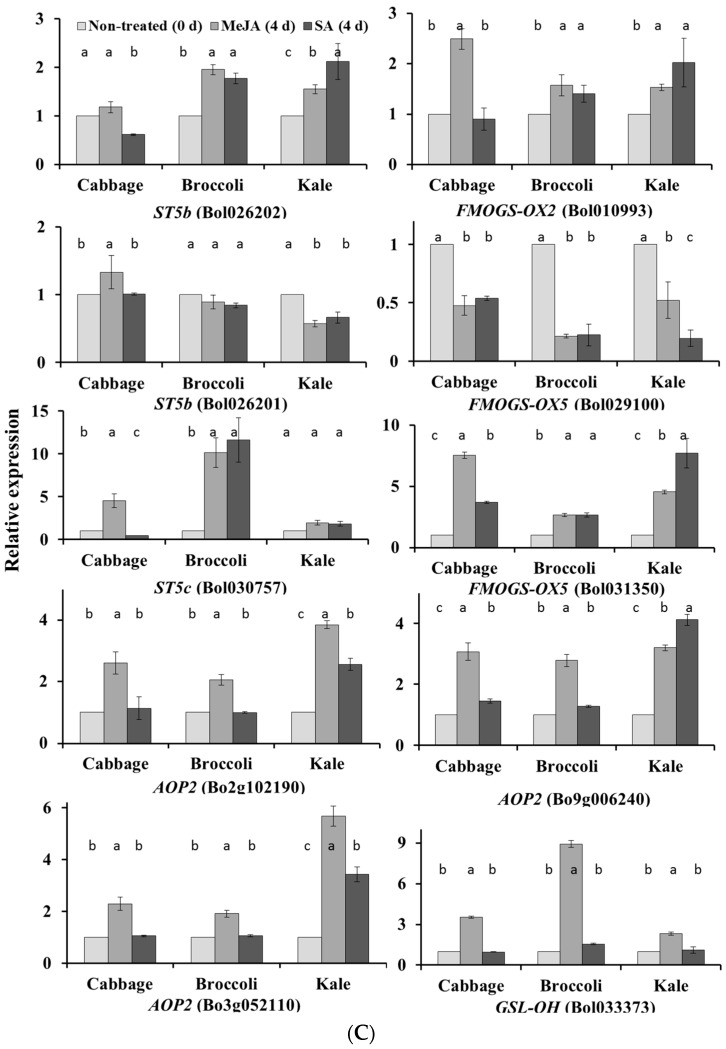

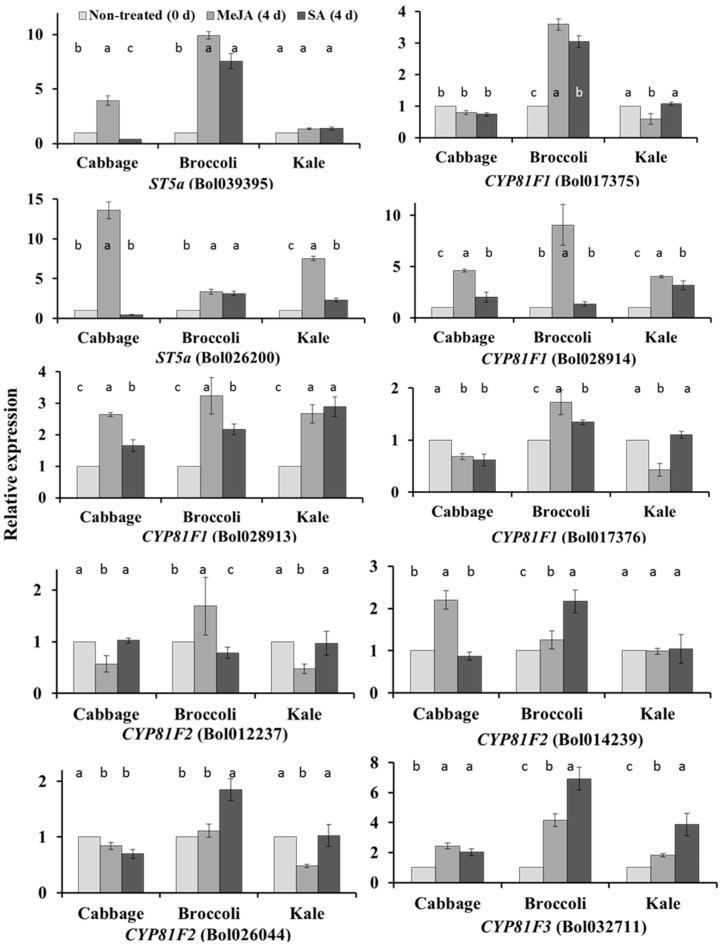

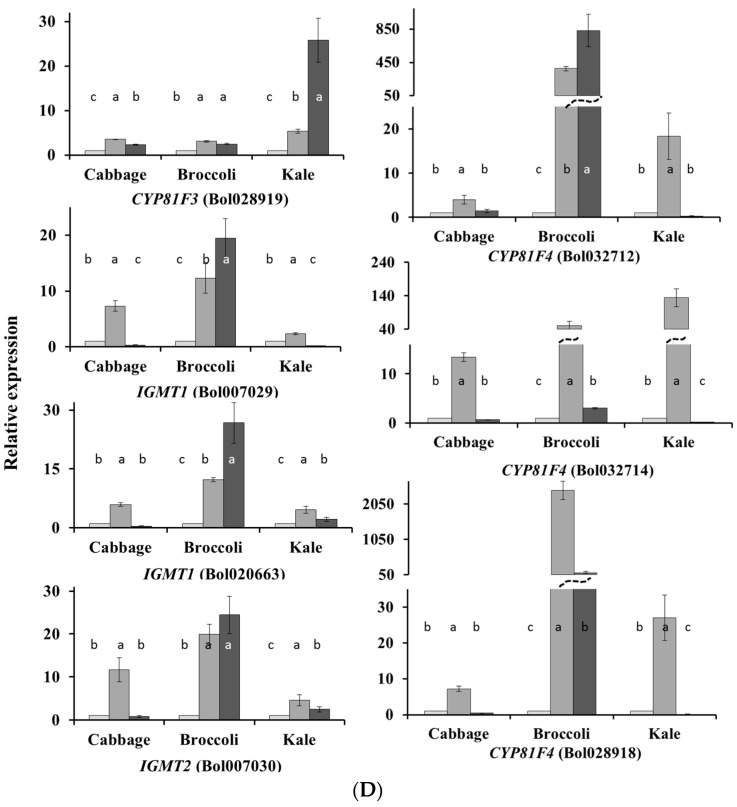
Quantitative PCR analyses of the expression of glucosinolate biosynthesis genes under the exogenous application of MeJA and SA. Expression was normalized to that of *actin* and the values in control plants were set to 1. Each data point is the average for each of the three biological replicates with three technical replicates against each biological replicate. Vertical bars indicate standard deviation of the means. Different letters indicate statistically significant difference (*p* < 0.01). (**A**) Relative expression analyses of five aliphatic transcription factor-related genes; (**B**) Relative expression analyses of six indolic transcription factor-related genes; (**C**) Relative expression analyses of 10 aliphatic glucosinolate biosynthesis-related genes; (**D**) Relative expression analyses of 17 indolic glucosinolate biosynthesis-related genes.

We carried out qPCR analysis of 38 glucosinolate biosynthesis-related genes from both aliphatic and indolic glucosinolate pathways (Figure 1). The results revealed that exogenous application of phytohormones affect the biosynthetic pathways in a variety-dependent manner. For example, the *MYB28* gene Bol036743 was upregulated in broccoli leaves under MeJA treatment (*p* < 0.01, Figure 2A and Figure S1), whereas *MYB28* genes Bol007795 in broccoli and Bol036286 in kale leaves were downregulated under both MeJA and SA treatment (*p* < 0.01 for both genes, Figure 2A and Figure S1). *MYB51* genes Bol013207 and Bol030761 were upregulated in kale under SA treatment and *MYB122* gene Bol026204 was upregulated in broccoli leaves under MeJA treatment (Figure 2B and Figure S1). Among the aliphatic glucosinolate biosynthesis-related genes, *FMOGS-OX2* (Bol010993) and *FMOGS-OX5* (Bol031350) were upregulated in cabbage leaves under MeJA treatment (Figure 2C and Figure S1). *CYP81F1* gene Bol017375 was upregulated only in broccoli under both MeJA and SA treatment (Figure 2C and Figure S1). *CYP81F4* genes Bol032712, Bol032714 and Bol028918 were downregulated under SA treatment in kale but upregulated in broccoli (Figure 2D and Figure S1). The more than 20-fold increase in expression of *CYP81F3* gene Bol028919 in kale leaves under SA treatment was also remarkable (Figure S1). Broccoli was the most responsive to exogenous treatments in terms of the relative expression of both aliphatic and indolic transcription factor-related genes compared to cabbage and kale (Figure 2B and Figure S1).

### 2.2. Subspecies-Independent Effects of Exogenous MeJA or SA on Glucosinolate Biosynthesis Gene Expression

In addition to subspecies-specific gene expression, some genes were upregulated in all three subspecies under MeJA or SA treatment. For example, *MYB34* genes Bol017062 and Bol007760 were notable, as they alone out of six indolic transcription factor-related genes, were upregulated in all three subspecies under MeJA treatment (*p* < 0.01 for both genes, Figure 2B and Figure S1). The level of increase ranged between 2- and 16-fold (Figure 2B). The *FMOGS-OX5* gene Bol029100 was downregulated in all three subspecies. The majority of the indolic glucosinolate biosynthesis genes were induced by either MeJA or SA treatment (Figure 2D and Figure S1). The level of expression of *CYP81F4* genes Bol032712, Bol032714 and Bol028918 were remarkably high (376-, 50- and 2434-fold upregulated in broccoli, respectively) in all three subspecies under MeJA treatment (Figure 2D and Figure S1).

### 2.3. Subspecies-Specific Glucosinolate Accumulation under MeJA or SA Treatment

HPLC analysis of leaves of the three subspecies detected eight different glucosinolates: five aliphatic glucosinolates, namely glucoiberin (GIB), progoitrin (PRO), glucoraphanin (GRA), sinigrin (SIN), and gluconapin (GNA), as well as three indolic glucosinolates: glucobrassicin (GBS), methoxyglucobrassicin (MGBS), and neoglucobrassicin (NGBS) (Table 1). Both MeJA and SA treatment increased the content of aliphatic GIB, PRO, SIN and indolic NGBS significantly in cabbage leaves (Table 1). SA treatment significantly increased the content of GNA in cabbage and that of GIB, GRA and MGBS in broccoli (Table 1). MeJA treatment significantly increased GBS in all three subspecies, SIN and NGBS in broccoli and NGBS in kale (Table 1). Notably, the content of all aliphatic glucosinolates in kale remained unaffected by exposure to exogenous MeJA or SA (Table 1). The content of GBS was 11-, 5- and 18-fold increased in cabbage, broccoli and kale leaves, respectively, under exogenous MeJA treatment compared to control plants (Table 1). The same treatment increased the content of NGBS by 4-, 158- and 19-fold in the cabbage, broccoli and kale leaves, respectively (Table 1). The SA treatment also increased the NGBS content by 4-fold in kale leaves (Table 1).

### 2.4. Associations between Glucosinolate Contents and Gene Expression

#### 2.4.1. MYB34, ST5a and CYP81 Gene Expression Is Related to GBS and NGBS Accumulation

Principal component analysis (PCA) indicated a probable association between indolic biosynthesis gene expression and glucosinolate contents (Table 2). PC2 coefficients explained 15% of the variation in the data, suggesting an association between higher GBS and NGBS contents and upregulation of *ST5a* (Bol026200), *CYP81F1* (Bol028913), *CYP81F1* (Bol028914), *CYP81F4* (Bol032714), *CYP81F4* (Bol028918) and downregulation of *CYP81F2* (Bol026044) (Table 2). This PC accounted for significant treatment differences, where MeJA-treated samples had the largest and positive PC scores and both the control and SA-treated samples had negative PC scores (Table 2; see also Figure 2D). PC2 in the PCA between indolic glucosinolate content and expression level of indolic biosynthesis transcription factor genes also demonstrated an association between GBS content and *MYB34* (Bol017062) expression with a significant treatment difference (Table S1). PC2 scores for that PC indicated that this variation is largely because of MeJA treatment (Table S1). PC1 and PC3, respectively explained 41.5% and 12.7% of the data variation between broccoli and the other two subspecies (Table 2). Exogenous MeJA treatment increased GBS most remarkably in cabbage and kale and NGBS in broccoli and that variation is largely explained by a negative relationship between GBS and NGBS content in PC3 (Table 1 and Table 2).

#### 2.4.2. CYP81F3 Gene Expression Is Related to MGBS Accumulation under SA Treatment

Similar to NGBS, accumulation of MGBS was subspecies specific (Table 1). The cabbage leaves contained no detectable MGBS (Table 1). Exogenous SA increased the MGBS content in broccoli leaves compared to control plants (Table 1). PC5 indicated a positive relationship between MGBS content and upregulation of *CYP81F3* genes in broccoli leaves under SA treatments (Table 2, Figure 2D).

#### 2.4.3. Expression of ST5c and FMOGS-OX5 Genes in Cabbage Is Related to Accumulation of Aliphatic Glucosinolates

We observed enhanced biosynthesis of GIB and a few other aliphatic glucosinolates in cabbage under both MeJA and SA treatment (Table 1). The GIB and GRA contents had similar relationships to the expression of transcription factor-related and aliphatic biosynthesis genes (Tables S2 and S3). The higher content of GIB and other aliphatic glucosinolates was associated with variation in expression levels of ST5c (Bol030757) and FMOGS-OX5 genes in treated cabbage plants compared to controls (Figure 2C).

### 2.5. Natural Variation in Glucosinolate Contents and Gene Expression

#### 2.5.1. Glucosinolate Accumulation and Gene Expression in Kale Leaves

**Figure 3:**
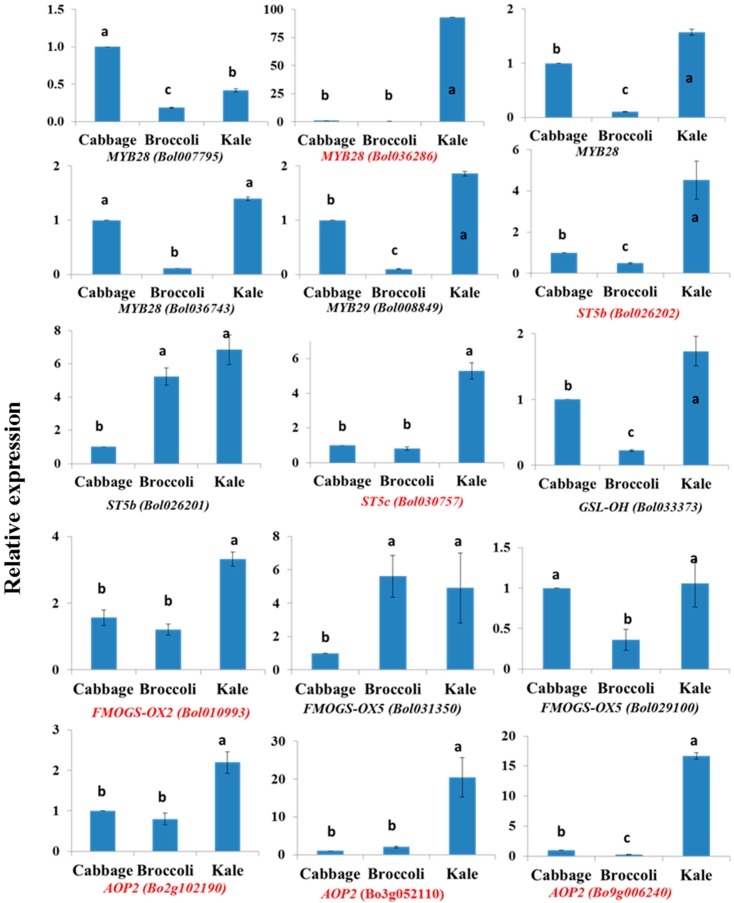
Subspecies variation in aliphatic glucosinolate biosynthesis-related gene expression in the control cabbage, broccoli and kale leaves. Vertical bars indicate standard deviation of means. Different letters indicate statistically significant differences between subspecies at 1% level of significance in one-way ANOVA. Genes with red letters were highly expressed only in kale.

In control plants, kale leaves had remarkably higher contents of PRO, SIN and GNA compared to cabbage and broccoli, revealing significant subspecies variation (Figure S3 and Table 3). Kale leaves also contained significantly more MGBS whereas cabbage leaves had higher contents of GIB and GRA (Figure S3). The PRO content in kale leaves was 13- and 72-fold higher compared to cabbage and broccoli leaves, respectively (Table 3). GNA content was 53-fold higher in kale leaves compared to cabbage (Table 3), whereas broccoli leaves contained no detectable GNA (Table 1). Among the aliphatic transcription factor-related genes, *MYB28* (Bol036286) showed 93- and 253-fold higher expression in kale leaves compared to cabbage and broccoli (Table 3, Figure 3). The expression of *ST5b*, *ST5c* and *AOP2* genes was 2.2- to 20.5-fold and 1.3- to 66-fold greater in kale than in cabbage and broccoli, respectively (Table 3). In addition, *FMOGS-OX5* (Bol031350) and *GSL-OH* (Bol033373) were expressed 4.9- and 7.8-fold more highly in kale leaves compared to cabbage and broccoli, respectively (Table 3, Figure 3).

#### 2.5.2. Cabbage Leaves Have Higher GIB and GRA Contents

GIB and GRA were at the highest levels in cabbage leaves compared to broccoli and kale leaves (Table 3, Figure S3). In addition, *MYB28* (Bol007795) had significantly higher expression in cabbage compared to broccoli and kale (Figure 3).

Among the indolic glucosinolates, GBS was present at the highest levels in cabbage leaves, at 2.24- and 2.6-fold higher than in broccoli and kale, but no MGBS was detected in cabbage (Figure S3 and Table 4). Kale leaves had 17-fold more MGBS compared to broccoli, whereas the broccoli leaves contained 4.3- and 2.5-fold more NGBS compared to cabbage and kale, respectively (Figure S3 and Table 4). *MYB34* (Bol007760, Bol036262), *MYB51* (Bol013207) and *MYB122* (Bol026204) showed more than two-fold higher expression in cabbage compared to the other two subspecies (Table 4). *CYP81F4* (Bol032712) and *CYP81F4* (Bol032714) had 2.7- and 316-fold higher transcript accumulation in cabbage compared to broccoli (Table 4). By contrast, *ST5a*, *CYP81F2*, *CYP81F3*, *IGMT1* and *IGMT2* showed higher expression in broccoli leaves compared to cabbage and kale (Table 4). *CYP81F1* showed much higher relative expression in kale leaves compared to broccoli and cabbage (Table 4).

## 3. Discussion

The present study investigated the relative expression of 38 genes related to glucosinolate biosynthesis and measured glucosinolate contents in cabbage, broccoli and kale leaves under the exogenous treatment of MeJA and SA. Both glucosinolate accumulation and expression level of biosynthetic genes revealed subspecies-specific variations in *B. oleracea*.

### 3.1. Subspecies-Specific Response to Exogenous MeJA and SA Application

The effects of exogenous MeJA and SA application have been widely studied in different species and subspecies of the Brassicales order. The previous studies indicated that exogenous MeJA application enhances the production of indolic and aromatic glucosinolates in leaves of *Arabidopsis thaliana* [43,44,45,46], *B. napus* [21], *B. juncea* [39] and *B. rapa* [22], as well as in different subspecies of *B. oleracea*, for example, in broccoli leaves [25,47,48], turnip [40], and in cauliflower curds [25]. The MeJA and SA phytohormones in *B. oleracea* subspecies each enhanced accumulation of particular glucosinolates in a variety-dependent manner. In this study, under the exogenous application of MeJA, the contents of leaf indolic GBS and NGBS were enhanced in all three subspecies (Figure 3). However, the fold-increase of a particular glucosinolate in the edible organs might vary between subspecies compared to leaf tissues. In previous studies, increase of GBS and NGBS was approximately 2-fold and 3-fold higher, respectively, in cauliflower curds [25] compared to kale leaf tissues [27]. A notably higher accumulation of the indolic MGBS in broccoli under SA application confirmed that MeJA or SA have subspecies-specific effects towards the accumulation of that particular glucosinolate (Table 1). Other than indolic GBS and NGBS, 250 μM MeJA treatment four days prior to harvest increased the content of aliphatic glucoraphanin (GRA) and aromatic gluconasturtiin (GST) in broccoli [28,49].

In contrast to some other previous studies, e.g., Ku et al. [28] and Liu et al. [49], this study recorded an enhanced accumulation of the aliphatic glucosinolates GIB, PRO, SIN in cabbage under both MeJA and SA treatment and in broccoli under SA treatment. Baenas et al. [50] reported significantly increased GIB content in broccoli sprouts under 250 μM MeJA treatments but no significant change under a similar dose of SA. In another subspecies of *B. oleracea* var. *alboglabra* Bailey (Chinese kale), MeJA treatment increased indolic GBS and NGBS but SA treatment increased aliphatic GNA and SIN [40], suggesting that the signaling response of a particular *B. oleracea* subspecies is also elicitor-specific.

### 3.2. Association between Glucosinolate Accumulation and Gene Expression under Exogenous MeJA and SA

In this study, 36 out of 38 examined genes, the exceptions being *CYP81F1* (Bol017376) and *CYP81F2* (Bol012237), showed significant treatment variation in terms of relative expression under the exogenous application of MeJA and SA (Figure S1). Generally, increased gene expression of only one or a few genes under a phytohormone treatment was associated with higher glucosinolate biosynthesis. Guo et al. [44] conducted expression profiling of selected transcription factor and glucosinolate biosynthesis genes, revealing that some *MYB* transcription factor and glucosinolate biosynthesis genes are highly induced under combined application of JA and glucose. Our current study suggested that both indolic biosynthesis transcription factor-related genes and indolic biosynthesis genes themselves are largely regulated by JA-mediated pathways, leading to higher indolic glucosinolate production under MeJA treatment. In *Arabidopsis*, MeJA was reported to induce some *CYP* genes that enhanced the indolic glucosinolate production by 3- to 4-fold [24]. However, in some recent studies the *BjuCYP83A1* and *CYP79F1* genes were found to regulate aliphatic glucosinolate biosynthesis in *B. juncea* [51,52].

One of the remarkable observations in this study is the striking increase, up to 2435-fold, of *CYP81F4* genes under MeJA application in all three subspecies (Figure 2D; [24]). The corresponding increase in GBS in under the same treatment indicated that the upregulation of *CYP81F4* might have an association with enhanced GBS biosynthesis under exogenous MeJA application. In *B. juncea*, Augustine and Bisht [39] reported a 9-fold increase in GBS at 48 h of MeJA treatment. When the *Arabidopsis* indolic glucosinolate biosynthesis pathway was engineered into *Nicotiana benthamiana*, *CYP81F4* was found to be the key gene responsible for the production of 1-methoxy-3-indolylmethyl glucosinolate from the initial 3-indolylmethyl glucosinolate (Figure 2D; [53]). *CYP81F4* has been suggested to have a significant role in the conversion of GBS to NGBS (Figure 2D, Table 2; [24,26]). In our study, the increase in GBS and NGBS in all three subspecies (Table 1) indicated that while *CYP81F4* likely functions in conversion of GBS to NGBS, some other enzymes involved might be induced by the transcription factors that induce biosynthesis of GBS. PC2 in Table S1, for the two highest coefficients of GBS content and *MYB34* expression indicated that higher GBS biosynthesis under MeJA treatment is possibly induced by *MYB34* (Figure 2B). Analysis of knockdown mutants in the presence of JA and SA along with other signaling compounds indicated that *MYB34* is a key regulator in JA signaling and *MYB51* is the central regulator in SA signaling, whereas *MYB122* plays a minor role in JA-induced glucosinolate biosynthesis [54]. However, in this study, high upregulation of *MYB51* genes Bol013207 and Bol030761 and *CYP81F3* (Bol028919) in kale leaves under SA treatment with a corresponding increase in NGBS content only in the kale indicated that the effect of SA is subspecies specific (Figure 2B,D, Table 1). *CYP81F* genes are involved in methoxylation that converts GBS to 4-methoxy-GBS, which has antifungal properties [55,56]. In addition, experimental evidence suggests that *CYP81F3*, similar to *CYP81F2*, catalyzes the conversion of I3M to 4OHI3M [53,57]. Such conversion might enhance NGBS biosynthesis in kale under SA treatment.

The observed increase in contents of GIB and some other aliphatic glucosinolate under MeJA treatment in cabbage was consistent with the approximately 67% increase in *B. juncea* seedlings after 24 h of MeJA treatment reported by Augustine and Bisht [39]. Under both MeJA and SA treatment, *MYB29* trans-activates the biosynthesis gene *ST5b* and also induces expression of transcription factors that upregulate the gene encoding the second enzyme in the glucosinolate biosynthesis pathway [54]. *MYB28* (Bol017019, Bol036743) and *FMOGS-OX5* (Bol031350) showed the highest level of relative expression in cabbage compared to control (Figure 2A,C). A significantly higher level of upregulation of *CYP81F4* gene in broccoli and kale compared to cabbage (Figure 2D) might be associated with higher accumulation of NGBS in those two subspecies.

### 3.3. Natural Variation in Glucosinolate Biosynthesis and Gene Expression at the Sub-Species Level

In a recent study with 25 kale varieties collected from three diverse geographical locations indicated that kale leaves are rich in aliphatic glucosinolates compared to reference broccoli but the wild landraces contained comparatively less glucosinolates compared to its commercial varieties [58]. Similarly, we observed a higher content of aliphatic glucosinolates PRO, SIN and GNA in the leaves of the control kale plants, indicating a natural subspecies-related variation in glucosinolate contents (Table 1). However, the corresponding greater relative expression of *MYB28* (Bol036286), *ST5b*, *ST5c* and *AOP2* genes indicated that the regulation of the aliphatic glucosinolate biosynthesis pathway varies between *B. oleracea* subspecies (Figure 3). In a recent study, expression of the kale BoMYB29 protein in Arabidopsis enhanced expression of aliphatic glucosinolate biosynthesis genes and simultaneously enhanced content of GRA, indicating that kale *BoMYB29* gene is a key regulator of methylsulphinyl glucosinolate biosynthesis [59]. Yi et al. [42] reported variation in glucosinolate content in the edible organs of four different subspecies of *B. oleracea*. Our results show that similar conclusions can be drawn for indolic glucosinolate biosynthesis based on contents and simultaneous expression of related genes (Table 2 and Table 4). Thus, a comparatively higher accumulation of GBS in cabbage might be associated with higher level of expression of *MYB34*, *MYB51* (Bol013207) and *MYB122* genes; a comparatively higher accumulation of MGBS in kale might be associated with relatively higher upregulation of *ST5a* (Bol026202), *IGMT1* and *IGMT2* genes; and a comparatively higher accumulation of NGBS in broccoli might be associated with *CYP81F1* genes (Figure 2, Table 2).

Other notable observations in this study were: (i) the broccoli leaves unusually measured higher amount of PRO and SIN (Table 1) which were often absent or trace in amount in previous studies [60,61] that could explain both genotypic and environmental variation; (ii) absence of GNA in broccoli (Table 1) might be associated with deletion of the enzyme sitting on the pathway branching point; (iii) in cabbage, missing MGBS might be a consequence of deletion of one of IGMT1 or IGMT2 enzymes. Three *AOP2* genes in this study were relatively less expressed in broccoli compared to cabbage and kale but among them Bo9g006240 was the least expressed gene (Figure 3) that might have been regulated biosynthesis of GNA in broccoli. The evolutionary or ecological reasons for the sub-species specific variation in glucosinolate content of *B. oleracea* species and species-specific responses to hormone treatments is a subject of further investigation whereas such evolutionary variation to date has been discussed at the species level in *Brassicaceae* [42]. The genes identified herein to be associated with glucosinolate accumulation are strong candidates to be exploited for selective accumulation of desired glucosinolates in *B. oleracea* subspecies.

## 4. Materials and Methods

### 4.1. Plant Materials and Growth Conditions

One variety from each of the three different subspecies of *B. oleracea L.* was selected for this study (Table S5). Seeds were obtained from Asia Seed Co., Ltd. (Seoul, Korea). The seeds were sown and seedlings were raised in garden soil mixture in a plant culture room at 25 ± 1 °C, 60% relative humidity and 80–120 μmol·m^−2^·s^−1^ light intensity. Seedlings were transferred at four weeks of age to a glasshouse. The plants were allowed to grow for another three months before imposing MeJA or SA treatments.

### 4.2. MeJA and SA Treatment

Two different treatments were applied on four-month-old, glasshouse-grown plants to study variation in the expression level of glucosinolate biosynthesis genes under exogenous treatment of MeJA and SA compared to “control” plants. The two treatments were 250 μM MeJA [25,28] and 800 μM SA [47], both of which were applied as solutions prepared in 0.1% Triton X-100. Control plants were sprayed with only 0.1% Triton X-100. Three plants of each variety were sprayed with 500 mL solution for each treatment. The plants were allowed to grow for four days before sample collection. Leaf samples were chosen for subspecies comparison as the level of glucosinolate-related gene expression was reported to be organ-specific [62,63]. The relative expression of treated samples for each subspecies was estimated by comparison to control plants. The content of glucosinolates was determined for both treated and control samples. Three biological replicates were destructively harvested each plant. Samples were frozen in liquid nitrogen and preserved at −80 °C until use for further analysis.

### 4.3. Primer Design for Glucosinolate Biosynthesis Genes

A total of 38 genes related to glucosinolate biosynthesis were selected for relative expression analysis under three treatments (Table 1). Among them, five and six genes respectively were aliphatic and indolic transcription factor-related, 10 and 17 were aliphatic and indolic glucosinolate biosynthesis-related genes. Primers were previously designed and their efficiency was tested by Robin et al. [64] (Table S6).

### 4.4. cDNA Synthesis and Real-Time Quantitative PCR (qPCR) Analysis

An RNeasy mini kit (Catalogue No. 74106, Qiagen, Valencia, CA, USA) was used to extract the total RNA of the leaf samples. cDNA was synthesized from the total RNA of each sample using a PrimeScript-based kit (Takara Bio, Inc., Shiga, Japan). iTaq™ SYBR^®^ Green Super-mix was used with ROX (Bio-Rad, Hercules, CA, USA) for real-time PCR. The reaction volume was 20 µL where 1 µL cDNA of 60 ng·µL^−1^ of each sample was used. The targeted DNA segment was amplified by denaturation at 95 °C for 10 min, followed by 40 cycles of amplification with denaturation at 94 °C for 20 s, annealing at 58 °C for 20 s and a final incubation and signal acquisition at 72 °C for 25 s (see Figure S4 for melting curves). The fluorescence was recorded for each sample at the end of each of the 40 cycles. LightCycler96 software was used for quantification (Cq) analysis (Roche, Mannheim, Germany). The relative expression level was calculated by the comparative 2^−ΔΔCt^ method [65]. The *actin* gene, GenBank accession No. JQ435879 that is expressed in all subspecies (Forward sequence: GTCGCTATTCAAGCTGTTCTCT; Reverse sequence: GAGAGCTTCTCCTTGATGTCTC) was the housekeeping gene [66]. A heat map was generated using Gene Cluster 3.0 [67] and Java TreeView [68] software using the log-transformed data of the relative expression level of glucosinolate biosynthesis-related genes under MeJA and SA treatment.

### 4.5. Desulfo-Glucosinolate Extraction for HPLC Analysis

The modified HPLC protocol, previously used by Yi et al. [42] and Robin et al. [64], was used to extract desulfo-glucosinolates from the treated and control leaf samples. Methanol treated frozen leaf tissue of about 10 g was powdered. The processed samples were initially incubated for 10 min at 70 °C. The samples were then kept at room temperature for 1 h. To eliminate structural components of the tissues and proteins the samples were then centrifuged at 10,000× *g* for 8 min at 4 °C. An anion-exchange chromatography was conducted with the collected supernatant. The process of centrifugation and anion-exchange chromatography was repeated twice and the supernatants from three steps were composited in a 5-mL tube. The pooled supernatants were the crude glucosinolates. To conduct a desulfation process 0.5 mL 50 mM barium acetate and 0.5 mL 50 mM lead acetate was mixed with the crude glucosinolates. In this step, the solution was centrifuged at 2000× *g* for 10 min. The samples were then loaded into a 0.5 M sodium acetate pre-equilibrated DEAE-Sephadex column. Prior to desulfation, the crude glucosinolate samples were rinsed with distilled water. Then, 250 μL aryl sulfatase was added to the column to commence desulfation process. The process was continued for 16 h before starting elution of desulfated glucosinolates with 1 mL distilled water. The eluted desulfo-glucosinolates was further purified by configuring at a high speed of 20,000× *g* for 4 min at 4 °C and filtering through a PTFE filter (13 mm, 0.2 μm, Advantec, Pleasanton, CA, USA). The desulfo-glucosinolate samples were then analyzed in a Waters 2695 HPLC system (Waters, Milford, MA, USA) equipped with a C_18_ column (Zorbax Eclipse XBD C_18_, 4.6 mm × 150 mm, Agilent Technologies, Palo Alto, CA, USA). Water and acetonitrile were used as mobile phase solvents during HPLC analysis of desulfated and purified glucosinolates. The purified desulfo-glucosinolates were detected using PDA 996 UV-visible detector (Waters) at a wavelength of 229 nm. A standard curve prepared for commercial sinigrin was used to quantify the detected glucosinolates. Mass spectrometry analysis (HPLC/MS, Agilent 1200 series, Agilent Technologies) facilitated the identification of individual glucosinolate molecules (HPLC/MS, Agilent 1200 series, Agilent Technologies) (Table S7).

### 4.6. Statistical Analysis

The relative expression level of each gene and contents of each glucosinolate in each subspecies under three treatments was analyzed via the generalized linear model (GLM) using MINITAB 15 statistical software (Minitab Inc., State College, PA, USA). Subspecies-to-subspecies variation was analyzed similarly. A principal component analysis was conducted taking either aliphatic or indolic glucosinolate content and the corresponding relative expression level of biosynthesis genes as a set of variables. PC scores obtained under three treatment and three subspecies combinations were also analyzed using a two-way ANOVA following the GLM procedure using MINITAB 15 statistical software. For separating means of three treatments under each subspecies a Tukey’s pairwise comparison test was conducted.

## 5. Conclusions

This study investigated the expression analysis of 38 glucosinolate biosynthesis-related genes and also estimated contents of glucosinolates under the exogenous treatment of MeJA and SA in *B. oleracea* subspecies. A subspecies-specific response in glucosinolate accumulation and gene expression was observed under MeJA or SA treatment. The increased accumulation of a particular glucosinolate was generally associated with upregulation of specific genes under MeJA or SA treatment. The deduced association between particular glucosinolate biosynthesis and gene expression improves our understanding of underlying genetics of glucosinolate accumulation under the exposure of biotic elicitors. The non-treated kale leaves measured strikingly higher content of PRO, SIN and GNA that was associated with higher expression level of aliphatic biosynthetic genes including *AOP2*, indicating natural variation in their glucosinolate biosynthesis.

## Figures and Tables

**Table 1:** Contents of aliphatic and indolic glucosinolates (µmol·g^−1^·DW) produced under the exogenous application of MeJA and SA in three *B. oleracea* subspecies.

Subspecies	Treatment	GIB	PRO	GRA	SIN	GNA	GBS	MGBS	NGBS
Cabbage	Control	0.38 ± 0.17 b	0.58 ± 0.66 b	0.60 ± 0.29 a	0.57 ± 0.25 b	0.06 ± 0.07 b	0.66 ± 0.20 b	bdl	0.01 ± 0.004 b
	MeJA	1.63 ± 0.54 a	2.33 ± 0.44 a	1.27 ± 0.41 a	1.58 ± 0.22 a	0.19 ± 0.06 ab	7.24 ± 3.16 a	bdl	0.04 ± 0.006 a
*p* (cabbage)	SA	1.40 ± 0.41 a	2.21 ± 0.18 a	1.75 ± 0.77 a	1.30 ± 0.13 a	0.25 ± 0.02 a	1.23 ± 1.23 b	bdl	0.04 ± 0.007 a
	Treatment	0.02	0.006	0.09	0.003	0.016	0.008		0.002
Broccoli	Control	0.20 ± 0.02 b	0.10 ± 0.027 a	0.09 ± 0.009 b	0.25 ± 0.062 ab	bdl	0.29 ± 0.29 b	0.01 ± 0.003 b	0.04 ± 0.023 b
*p* (broccoli)	MeJA	0.23 ± 0.015 ab	0.10 ± 0.006 a	0.08 ± 0.015 b	0.40 ± 0.083 a	bdl	1.46 ± 0.09 a	0.11 ± 0.016 ab	6.31 ± 2.07 a
	SA	0.28 ± 0.025 a	0.13 ± 0.036 a	0.14 ± 0.02 a	0.14 ± 0.065 b	bdl	0.20 ± 0.16 b	0.19 ± 0.087 a	0.11 ± 0.024 b
	Treatment	0.012	0.37	0.007	0.011		<0.001	0.016	0.001
Kale	Control	0.11 ± 0.025 a	7.54 ± 5.02 a	0.20 ± 0.087 a	4.11 ± 2.25 a	3.02 ± 1.63 a	0.25 ± 0.102 b	0.02 ± 0.002 a	0.02 ± 0.01 b
	MeJA	0.41 ± 0.438 a	6.20 ± 5.54 a	1.67 ± 2.35 a	6.37 ± 3.93 a	2.23 ± 1.12 a	4.52 ± 0.817 a	0.08 ± 0.027 a	0.38 ± 0.24 a
	SA	0.10 ± 0.058 a	5.48 ± 3.11 a	0.63 ± 0.735 a	2.89 ± 0.493 a	2.54 ± 1.43 a	0.45 ± 0.299 b	0.08 ± 0.055 a	0.07 ± 0.035 ab
*p* (kale)	Treatment	0.301	0.864	0.473	0.326	0.795	<0.001	0.14	0.041
*p* value	Subspecies	<0.001	<0.001	NS	<0.001	<0.001	0.005	<0.001	<0.001

Each data point is the average of three biological replicates ± standard deviation; *p*, probability values for statistical significance of treatment, subspecies and treatment × subspecies against each glucosinolate compound; NS, not significant; bdl, below detection limit. Different lower case letters indicate statistically significant differences (see Table S4 for the HPLC data).

**Table 2:** Principal component analysis for indolic glucosinolate contents and relative expression of genes in three subspecies of *B. oleracea* in control, MeJA-treated and SA-treated plants. PC, principal component; *p*, statistical significance.

**Variable**	**PC1**	**PC2**	**PC3**	**PC4**	**PC5**
GBS	−0.025	0.326	−0.416	0.081	−0.250
MGBS	0.253	0.031	0.030	−0.017	0.345
NGBS	0.150	0.344	0.344	−0.107	−0.044
*ST5a* (Bol026200)	0.078	0.310	0.446	0.164	−0.174
*ST5a* (Bol039395)	0.317	0.098	0.050	0.012	−0.250
*CYP81F1* (Bol028913)	0.199	0.259	−0.101	0.387	0.013
*CYP81F1* (Bol028914)	0.053	0.483	0.163	−0.125	0.171
*CYP81F1* (Bol017375)	0.305	−0.007	0.197	0.096	−0.165
*CYP81F1* (Bol017376)	0.245	−0.071	0.260	0.272	−0.219
*CYP81F2* (Bol012237)	0.155	−0.065	0.250	0.423	−0.147
*CYP81F2* (Bol014239)	0.210	−0.113	−0.321	−0.052	0.041
*CYP81F2* (Bol026044)	0.244	−0.285	−0.036	−0.125	0.230
*CYP81F3* (Bol032711)	0.284	−0.090	−0.103	−0.045	0.369
*CYP81F3* (Bol028919)	0.016	0.084	0.019	0.554	0.541
*CYP81F4* (Bol032712)	0.296	−0.082	−0.018	−0.058	−0.153
*CYP81F4* (Bol032714)	0.004	0.326	−0.182	−0.122	0.237
*CYP81F4* (Bol028918)	0.111	0.349	0.314	−0.344	0.066
*IGMT1* (Bol007029)	0.318	−0.070	−0.138	−0.143	−0.115
*IGMT1* (Bol020663)	0.304	−0.100	−0.152	−0.200	0.116
*IGMT2* (Bol007030)	0.331	0.003	−0.105	−0.057	−0.059
% variation explained	41.5	15.0	12.7	7.6	6.3
*p* (subspecies)	<0.01	0.49	<0.01	0.05	<0.01
*p* (treatment)	<0.01	<0.01	<0.01	0.46	<0.01
*p* (subspecies × treatment)	<0.01	0.019	<0.01	0.03	0.16
**Source of Variation**	**Mean PC Scores (±Sd)**				
Subspecies					
Cabbage	−1.26 ± 0.41	−0.06 ± 0.22	−0.57 ± 0.17	−0.01 ± 0.26	−0.58 ± 0.2
Broccoli	2.35 ± 0.41	−0.15 ± 0.22	0.77 ± 0.17	−0.47 ± 0.26	−0.01 ± 0.2
Kale	−1.09 ± 0.41	0.21 ± 0.22	−0.19 ± 0.17	0.48 ± 0.26	0.59 ± 0.2
Treatment					
Control	−1.57 ± 0.41	−1.11 ± 0.22	0.63 ± 0.17	−0.22 ± 0.26	−0.45 ± 0.2
MeJA	0.76 ± 0.41	1.96 ± 0.22	−0.61 ± 0.17	−0.02 ± 0.26	−0.30 ± 0.2
SA	0.81 ± 0.41	−0.84 ± 0.22	−0.01 ± 0.17	0.24 ± 0.26	0.75 ± 0.2
Subspecies × treatment					
Cabbage × Control	−1.61 ± 0.71	−1.09 ± 0.38	0.58 ± 0.30	−0.21 ± 0.45	−0.52 ± 0.35
Cabbage × MeJA	−0.31 ± 0.71	1.47 ± 0.38	−2.69 ± 0.30	0.24 ± 0.45	−1.03 ± 0.35
Cabbage × SA	−1.85 ± 0.71	−0.56 ± 0.38	0.38 ± 0.30	−0.05 ± 0.45	−0.20 ± 0.35
Broccoli × Control	−1.56 ± 0.71	−1.13 ± 0.38	0.65 ± 0.30	−0.23 ± 0.45	−0.43 ± 0.35
Broccoli × MeJA	3.81 ± 0.71	2.52 ± 0.38	2.47 ± 0.30	−0.25 ± 0.45	−0.36 ± 0.35
Broccoli × SA	4.82 ± 0.71	−1.85 ± 0.38	−0.81 ± 0.30	−0.93 ± 0.45	0.76 ± 0.35
Kale × Control	−1.54 ± 0.71	−1.12 ± 0.38	0.64 ± 0.30	−0.23 ± 0.45	−0.40 ± 0.35
Kale × MeJA	−1.21 ± 0.71	1.87 ± 0.38	−1.63 ± 0.30	−0.05 ± 0.45	0.48 ± 0.35
Kale × SA	−0.52 ± 0.71	−0.11 ± 0.38	0.39 ± 0.30	1.71 ± 0.45	1.69 ± 0.35

**Table 3:** Fold increase in relative expression of aliphatic glucosinolate transcription factor and biosynthesis genes and glucosinolate contents in kale leaves compared to cabbage and broccoli leaves for the control plants. Fold increase was calculated based on mean values obtained from three biological replicates.

Genes	Kale: Cabbage	Kale: Broccoli
**Aliphatic Transcription Factor-Related**		
*MYB28* (Bol007795)	0.4	2.3
*MYB28* (Bol036286)	93	253
*MYB28* (Bol017019)	1.6	15.6
*MYB28* (Bol036743)	1.4	12.9
*MYB29* (Bol008849)	1.9	19.1
**Aliphatic Biosynthesis-Related**		
*FMOGS-OX2* (Bol010993)	3.3	2.7
*FMOGS-OX5* (Bol029100)	1.1	2.9
*FMOGS-OX5* (Bol031350)	4.9	0.9
*GSL-OH* (Bol033373)	1.7	7.8
*ST5b* (Bol026202)	4.5	9.1
*ST5b* (Bol026201)	6.8	1.3
*ST5c* (Bol030757)	5.3	6.5
*AOP2* (Bo2g102190)	2.2	2.7
*AOP2* (Bo3g052110)	20.5	10
*AOP2* (Bo9g006240)	16.7	66
**Glucosinolate Compounds**		
GIB	0.3	0.6
PRO	13	72
GRA	0.3	2.2
SIN	7.2	17
GNA	53	-

**Table 4:** Upregulation of indolic glucosinolate transcription factor and biosynthesis genes in one subspecies compared to other two subspecies for the control plants.

Gene Name	Accession Number	GBS (Cabbage)		NGBS (Broccoli)		MGBS (Kale)	
		Broccoli	Kale	Cabbage	Kale	Cabbage	Broccoli
*MYB34*	Bol007760	2.5	20.4				
	Bol017062	1.0	2.7				
	Bol036262	3.4	2.7				
*MYB51*	Bol013207	2.0	3.0				
	Bol030761	0.3	1.9				
*MYB122*	Bol026204	2.2	3.1				
*ST5a*	Bol026200			3.5	6.5		
	Bol039395			1.9	1.3		
*CYP81F4*	Bol032712	2.7	1.0				
	Bol032714	316	0.6				
	Bol028918					1.44	1.4
*CYP81F1*	Bol017375					4.3	24
	Bol017376					2.8	35
	Bol028913					2.2	1.4
	Bol028914			3.2	3.8		
*CYP81F2*	Bol012237			1.2	1.95		
	Bol014239			2.5	2.1		
	Bol026044			1.5	1.9		
*CYP81F3*	Bol028919			2.6	1.4		
	Bol032711			1.5	1.9		
*IGMT1*	Bol007029			3.98	5.1		
	Bol020663			3.5	2.35		
*IGMT2*	Bol007030			1.8	3.97		
Content increased (fold)		2.24	2.6	4.3	2.54	α (infinity)	17

## Supplementary Materials

Supplementary materials can be accessed at: http://www.mdpi.com/1420-3049/21/10/1417/s1.

## Abbreviations

GIB: glucoiberin
PRO: progoitrin
GRA: glucoraphanin
SIN: sinigrin
GNA: gluconapin
GBS: glucobrassicin
MGBS: methoxyglucobrassicin
NGBS: neoglucobrassicin
3MTP: 3-methylthiopropyl glucosinolate (glucoiberverin)
4MTB: 4-methylthiobutyl glucosinolate (glucoerucin)
3MSOP: 3-(methylsulfinyl)propyl glucosinolate (glucoiberin)
4MSOB: 4-(methylsulfinyl)butyl GSL (glucoraphanin)
Sinigrin: allyl glucosinolate
3BTEY: 3-butenyl glucosinolate (Gluconapin)
4OHB: 4-hydroxybutyl glucosinolate (progoitrin)
I3M: 3-indolylmethyl glucosinolate (glucobrassicin)
4OHI3M: 4-hydroxy-3-indolylmethyl glucosinolate (4-hydroxyglucobrassicin)
1MOI3M: 1-methoxy-3-indolylmethyl glucosinolate (neoglucobrassicin)
4MOI3M: 4-methoxy-3-indolylmethyl glucosinolate (4-methoxyglucobrassicin)
IGM: indole GSL modifier
