# Supplementary material for: Exogenous Methyl Jasmonate and Salicylic Acid Induce Subspecies-Specific Patterns of Glucosinolate Accumulation and Gene Expression in Brassica oleracea L
Source: Molecules. 2016 Oct 24;21(10):1417. doi: 10.3390/molecules21101417 (PMC6273115; doi:10.3390/molecules21101417)
Supplement: Supplementary file 1 [file molecules-21-01417-s001.pdf]

Supplementary Materials: Exogenous Methyl Jasmonate and Salicylic Acid Induce Subspecies-Specific Patterns of Glucosinolate Accumulation and Gene Expression in *Brassica oleracea* L.

Go-Eun Yi, Arif Hasan Khan Robin, Kiwoung Yang, Jong-In Park, Byung Ho Hwang and Ill-Sup Nou

Table S1. Principal component analysis (PCA) for glucosinolate contents and relative expression of indolic transcription factor-related genes in three subspecies of *B. oleracea* in control, MeJA-treated and SA-treated plants. PC, principal component; *P*, statistical significance.

Variable	PC1	PC2	PC3	PC4	PC5
GBS	−0.023	0.560	−0.079	−0.506	0.509
MGBS	0.251	−0.018	−0.327	0.706	0.499
NGBS	0.427	0.174	0.138	0.122	−0.468
MYB34 (Bol017062)	0.236	0.536	−0.213	0.068	−0.020
MYB34 (Bol007760)	0.445	0.283	0.104	0.024	−0.187
MYB34 (Bol036262)	0.221	−0.226	0.650	−0.045	0.434
MYB51 (Bol013207)	0.307	−0.352	−0.426	−0.401	−0.093
MYB51 (Bol030761)	0.384	−0.333	−0.336	−0.227	0.185
MYB122 (Bol015939)	0.459	−0.052	0.308	−0.102	0.079
%variation explained	42.8	19.5	14.4	10.1	6.6
p (subspecies)	<0.01	0.46	<0.01	<0.01	0.64
p (treatment)	<0.01	<0.01	<0.01	0.08	0.08
p (subspecies × treatment)	<0.01	0.04	<0.01	<0.01	<0.01
**Source of Variation**	**Mean PC Scores (±Sd)**				
Subspecies					
Cabbage	−0.83 ± 0.28	0.12 ± 0.17	0.54 ± 0.17	−0.64 ± 0.16	0.10 ± 0.14
Broccoli	1.33 ± 0.28	−0.17 ± 0.17	0.24 ± 0.17	0.63 ± 0.16	0.001 ± 0.14
Kale	−0.49 ± 0.28	0.05 ± 0.17	−0.78 ± 0.17	0.01 ± 0.16	−0.10 ± 0.14
Treatment					
Control	−0.87 ± 0.28	−0.60 ± 0.17	0.72 ± 0.17	−0.06 ± 0.16	−0.23 ± 0.14
MeJA	1.05 ± 0.28	1.48 ± 0.17	−0.15 ± 0.17	−0.22 ± 0.16	−0.01 ± 0.14
SA	−0.17 ± 0.28	−0.88 ± 0.17	−0.56 ± 0.17	0.29 ± 0.16	0.24 ± 0.14
Subspecies × treatment					
Cabbage × Control	−0.91 ± 0.48	−0.55 ± 0.31	0.75 ± 0.29	−0.21 ± 0.27	−0.25 ± 0.25
Cabbage × MeJA	−0.74 ± 0.48	1.53 ± 0.31	0.24 ± 0.29	−1.26 ± 0.27	0.64 ± 0.25
Cabbage × SA	−0.84 ± 0.48	−0.60 ± 0.31	0.62 ± 0.29	−0.45 ± 0.27	−0.08 ± 0.25
Broccoli × Control	−0.86 ± 0.48	−0.62 ± 0.31	0.71 ± 0.29	−0.02 ± 0.27	−0.24 ± 0.25
Broccoli × MeJA	4.40 ± 0.48	0.80 ± 0.31	0.59 ± 0.29	0.37 ± 0.27	−0.77 ± 0.25
Broccoli × SA	0.45 ± 0.48	−0.71 ± 0.31	−0.58 ± 0.29	1.55 ± 0.27	1.02 ± 0.25
Kale × Control	−0.84 ± 0.48	−0.62 ± 0.31	0.68 ± 0.29	0.02 ± 0.27	−0.19 ± 0.25
Kale × MeJA	−0.50 ± 0.48	2.10 ± 0.31	−1.30 ± 0.29	0.22 ± 0.27	0.08 ± 0.25
Kale × SA	−0.13 ± 0.48	−1.32 ± 0.31	−1.73 ± 0.29	0.29 ± 0.27	−0.19 ± 0.25

Table S2. Principal component analysis (PCA) for glucosinolate contents and relative expression of aliphatic transcription factor-related genes in three subspecies of *B. oleracea* in control, MeJA-treated and SA-treated plants. PC, principal component; *p*, statistical significance.

**Variable**	**PC1**	**PC2**	**PC3**	**PC4**	**PC5**
GIB	−0.130	−0.287	0.570	0.268	0.228
PRO	0.486	0.090	−0.048	0.315	0.182
GRA	0.021	−0.187	0.700	0.040	−0.017
SIN	0.458	0.088	0.002	0.419	0.003
GNA	0.487	0.159	0.037	0.132	0.075
MYB28 (Bol007795)	0.283	0.048	0.083	−0.637	0.578
MYB28 (Bol036286)	−0.329	0.069	−0.169	0.277	0.750
MYB28 (Bol017019)	−0.229	0.565	0.132	0.125	0.033
MYB28 (Bol036743)	−0.205	0.561	0.138	0.159	−0.036
MYB29 (Bol008849)	0.131	0.448	0.331	−0.334	−0.099
%variation explained	34.4	22.5	16.3	9.9	7.2
p (subspecies)	<0.01	<0.01	<0.01	0.47	0.18
p (treatment)	0.24	<0.01	<0.01	<0.01	0.11
p (subspecies × treatment)	0.02	<0.01	0.41	<0.01	<0.01
**Source of Variation**	**Mean PC Scores (±Sd)**				
Subspecies					
Cabbage	−0.65 ± 0.21	−0.99 ± 0.19	0.68 ± 0.23	0.15 ± 0.18	0.26 ± 0.17
Broccoli	−1.50 ± 0.21	0.55 ± 0.19	−0.77 ± 0.23	−0.16 ± 0.18	−0.16 ± 0.17
Kale	2.16 ± 0.21	0.43 ± 0.19	0.09 ± 0.23	0.01 ± 0.18	−0.09 ± 0.17
Treatment					
Control	0.16 ± 0.21	−0.74 ± 0.19	−0.78 ± 0.23	−0.40 ± 0.18	0.28 ± 0.17
MeJA	−0.29 ± 0.21	0.85 ± 0.19	0.56 ± 0.23	0.68 ± 0.18	−0.05 ± 0.17
SA	0.13 ± 0.21	−0.11 ± 0.19	0.21 ± 0.23	−0.27 ± 0.18	−0.23 ± 0.17
Subspecies × treatment					
Cabbage × Control	−0.58 ± 0.37	−1.09 ± 0.33	−0.32 ± 0.41	−0.66 ± 0.31	0.25 ± 0.30
Cabbage × MeJA	−0.88 ± 0.37	−0.68 ± 0.33	1.06 ± 0.41	092 ± 0.31	−0.04 ± 0.30
Cabbage × SA	−0.50 ± 0.37	−1.20 ± 0.33	1.29 ± 0.41	0.19 ± 0.31	0.57 ± 0.30
Broccoli × Control	−0.72 ± 0.37	−0.83 ± 0.33	−1.11 ± 0.41	−1.00 ± 0.31	0.07 ± 0.30
Broccoli × MeJA	2.52 ± 0.37	2.97 ± 0.33	−0.22 ± 0.41	0.48 ± 0.31	0.41 ± 0.30
Broccoli × SA	−1.27 ± 0.37	−0.46 ± 0.33	−0.97 ± 0.41	0.03 ± 0.31	−1.00 ± 0.30
Kale × Control	1.78 ± 0.37	−0.29 ± 0.33	−0.90 ± 0.41	0.45 ± 0.31	0.52 ± 0.30
Kale × MeJA	2.51 ± 0.37	0.26 ± 0.33	0.83 ± 0.41	0.63 ± 0.31	−0.53 ± 0.30
Kale × SA	2.18 ± 0.37	1.33 ± 0.33	0.33 ± 0.41	−1.06 ± 0.31	−0.27 ± 0.30

Table S3. Principal component analysis (PCA) for glucosinolate contents and relative expression of aliphatic biosynthesis-related genes in three subspecies of *B. oleracea* in control, MeJA-treated and SA-treated plants. PC, principal component; *p*, statistical significance.

**Variable**	**PC1**	**PC2**	**PC3**	**PC4**	**PC5**
GIB	0.004	−0.136	−0.550	−0.142	−0.117
PRO	0.169	0.463	0.078	−0.275	0.086
GRA	0.154	−0.022	−0.498	0.201	−0.254
SIN	0.206	0.445	0.067	−0.043	0.054
GNA	0.222	0.436	0.100	−0.131	0.032
ST5b(Bol026201)	−0.120	−0.145	−0.337	−0.232	0.685
ST5b(Bol026202)	0.263	−0.224	0.327	−0.020	0.144
ST5c(Bol030757)	0.115	−0.374	0.305	−0.162	0.128
AOP2(190)	0.382	0.013	−0.110	0.384	0.221
AOP2(110)	0.386	0.051	−0.095	0.430	0.128
AOP2(240)	0.390	−0.059	−0.106	0.009	0.242
GSL-OH(Bol033373)	0.143	−0.283	0.170	0.219	0.006
FMOGS-OX5(Bol029100)	−0.296	0.202	−0.120	0.065	0.439
FMOGS-OX2(Bol010993)	0.309	−0.193	−0.019	−0.449	0.095
FMOGS-OX5(Bol031350)	0.330	−0.061	−0.199	−0.421	−0.280
%variation explained	31.0	21.0	15.4	8.1	6.4
p (subspecies)	<0.01	<0.01	<0.01	0.29	0.72
p (treatment)	<0.01	<0.001	0.72	0.11	<0.01
p (subspecies × treatment)	0.03	0.02	<0.01	<0.01	0.79
**Source of Variation**	**Mean PC Scores (±Sd)**				
Subspecies					
Cabbage	−0.73 ± 0.27	−0.36 ± 0.24	−1.42 ± 0.23	−0.26 ± 0.21	−0.12 ± 0.21
Broccoli	−0.73 ± 0.27	−1.37 ± 0.24	1.06 ± 0.23	0.21 ± 0.21	0.12 ± 0.21
Kale	1.46 ± 0.27	1.73 ± 0.24	0.36 ± 0.23	0.04 ± 0.21	0.001 ± 0.21
Treatment					
Control	−1.90 ± 0.27	0.76 ± 0.24	0.07 ± 0.23	0.27 ± 0.21	0.55 ± 0.21
MeJA	1.59 ± 0.27	−0.54 ± 0.24	−0.15 ± 0.23	0.08 ± 0.21	0.06 ± 0.21
SA	0.30 ± 0.27	−0.21 ± 0.24	0.07 ± 0.23	−0.36 ± 0.21	−0.61 ± 0.21
Subspecies × treatment					
Cabbage × Control	−2.14 ± 0.47	0.04 ± 0.41	−0.43 ± 0.40	0.49 ± 0.37	0.34 ± 0.36
Cabbage × MeJA	1.14 ± 0.47	−1.23 ± 0.41	−1.91 ± 0.40	−1.31 ± 0.37	0.18 ± 0.36
Cabbage × SA	−1.20 ± 0.47	0.10 ± 0.41	−1.93 ± 0.40	0.03 ± 0.37	−0.90 ± 0.36
Broccoli × Control	−2.33 ± 0.47	−0.05 ± 0.41	0.19 ± 0.40	0.52 ± 0.37	0.55 ± 0.36
Broccoli × MeJA	0.66 ± 0.47	−2.34 ± 0.41	1.56 ± 0.40	0.53 ± 0.37	0.20 ± 0.36
Broccoli × SA	−0.53 ± 0.47	−1.72 ± 0.41	1.43 ± 0.40	−0.41 ± 0.37	−0.39 ± 0.36
Kale × Control	−1.23 ± 0.47	2.28 ± 0.41	0.47 ± 0.40	−0.18 ± 0.37	0.75 ± 0.36
Kale × MeJA	2.97 ± 0.47	1.95 ± 0.41	−0.11 ± 0.40	1.03 ± 0.37	−0.18 ± 0.36
Kale × SA	2.65 ± 0.47	0.96 ± 0.41	0.73 ± 0.40	−0.71 ± 0.37	−0.56 ± 0.36

Table S4 HPLC recorded contents of aliphatic and indolic glucosinolates (μmol·g^{-1} DW) for each of the three biological replicates of three *B. oleracea* subspecies.

Subspeceis	Treatment	Biological Replicate	GIB	PRO	GRA	SIN	GNA	GBS	MGBS	NGBS
Cabbage	Control	1	0.536355	1.291864	0.872016	0.862	0.139747	0.72379	0.0000	0.014813
		2	0.4	0.433606	0.620217	0.391582	0.032803	0.820132	0.0000	0.006809
		3	0.2	0	0.299955	0.459526	0	0.432882	0.0000	0.008033
	MeJA	1	2.222961	2.783915	1.725882	1.809928	0.118222	10.88554	0.0000	0.03591
		2	1.5	1.90798	0.935985	1.370142	0.229597	5.607139	0.0000	0.046511
		3	1.15847	2.296198	1.154994	1.57093	0.232905	5.220712	0.0000	0.036303
	SA	1	1.8	2.208253	2.636135	1.43105	0.234778	1.476323	0.0000	0.045432
		2	1.4	2.400424	1.276082	1.168948	0.245161	1.329414	0.0000	0.032782
		3	0.986168	2.034036	1.334795	1.29898	0.281532	0.897427	0.0000	0.031859
Broccoli	Control	1	0.1698	0.0841	0.0889	0.2815	0.0000	0.1278	0.0099	0.0445
		2	0.2107	0.1363	0.0999	0.2808	0.0000	0.6334	0.0154	0.0651
		3	0.2073	0.0936	0.0813	0.1737	0.0000	0.1194	0.0105	0.0179
	MeJA	1	0.2345	0.1021	0.0839	0.3230	0.0000	1.5536	0.1090	3.9393
		2	0.2478	0.0951	0.0851	0.4878	0.0000	1.3669	0.1318	7.2512
		3	0.2171	0.0894	0.0574	0.3867	0.0000	1.4716	0.1001	7.7452
	SA	1	0.3050	0.1618	0.1584	0.1968	0.0000	0.3838	0.2736	0.1226
		2	0.2597	0.0903	0.1371	0.0672	0.0000	0.1190	0.1900	0.1177
		3	0.2608	0.1312	0.1180	0.1411	0.0000	0.0881	0.0992	0.0787
Kale	Control	1	0.0919	4.6307	0.1565	2.9566	2.1658	0.3236	0.0182	0.0285
		2	0.1368	13.3345	0.3005	6.6997	4.9068	0.3015	0.0235	0.0108
		3	0.0946	4.6599	0.1417	2.6708	1.9893	0.1372	0.0193	0.0107
	MeJA	1	0.9180	0.0640	4.3761	2.7680	1.9533	5.3585	0.0774	0.5703
		2	0.1654	10.8301	0.4073	10.5710	3.4643	3.7344	0.0516	0.4629
		3	0.1520	7.7082	0.2265	5.7825	1.2785	4.4685	0.1052	0.1050
	SA	1	0.1620	9.0041	0.3120	3.2888	4.0675	0.7081	0.1381	0.1082
		2	0.0589	4.3043	1.4655	2.3362	2.2967	0.5198	0.0704	0.0589
		3	0.0645	3.1352	0.0985	3.0310	1.2408	0.1220	0.0301	0.0390

Table S5. *B. oleracea* subspecies and varieties/lines used in this study.

No.	Common Name	Variety	Sampling Site	Scientific Name
1	Cabbage	Ohganae	Leaf	*B. oleracea capitata*
2	Broccoli	Asiaseed 42	Leaf	*B. oleracea italica*
3	Kale	Majjangkale	Leaf	*B. oleracea acephala*

Table S6. Primer sequences of 38 glucosinolate biosynthetic-related genes used for relative expression analysis through qPCR under exogenous methyl jasmonate and salicylic acid application [1].

Gene Name	Accession Number	cDNA Size(bp)	Forward Primer Sequence	Reverse Primer Sequence	Product Size(bp)
			Transcription factor-related genes (11 genes)		
MYB28	Bol007795	558	CCACACCAGTTCAGAGAGGT	GGGAAATGGATCGAAGTCAGC	221
	Bol036286	615	GAAGGTAGCTTGAATGCTAATAC	ATTCATGTAGTGCTCCTCATTC	249
	Bol017019	426	GTTGCGGCTAAGGTCACTTCT	CAGAAGTAGCGTTGATCTCATGC	223
MYB29	Bol036743	426	CTTGGGCGCTGCTACATTAC	ATCGTTCTCCTCGTTGTGGT	241
	Bol008849	513	CGCCCAAGACTTCTGAGTT	TGATATTGCCCATGGAAGCTG	234
MYB34	Bol007760	843	TGAAGGAGGATGGCGTACTC	CAGTTCGTCCCGCCAAATTA	203
	Bol017062	951	AAGGTGGATGGCGTACTCTC	TGTGAGTGGTTGGATCGACA	279
MYB51	Bol036262	294	CCCCGAGTTCTTTAGCAACC	TCCAAGTCCAGATCGTCTTCT	198
	Bol013207	1002	CCAGAGATTCCAGAGAAGC	CAAGTCACACTGCTACTACTAC	233
	Bol030761	990	CAGACAACTATATCGAGTAACG	TATCATTAACGGTCATCTGG	274
MYB122	Bol026204	981	GACCATTCCGAGACATTGCC	GCATCGTGGATCATGTGGAG	284
			Aliphatic biosynthesis-related genes (10 genes)		
ST5b	Bol026201	1035	CCGAGCCGTCAGAATTCAAG	GCTATGGCGAAAGTGAGAGC	247
	Bol026202	1035	AAGCCTTGACTTTCGCCATC	ACTTCACAACTGAGTCCGGT	204
ST5c	Bol030757	1014	CCACGCCCAAAACTTCTTCA	TGAGTGGAGAAGAGCGTGTT	246
FMOGS-OX2	Bol010993	1386	GAGAAGGTATCCGAGCCACA	GTCCACTGCAAACAACGACT	200
FMOGS-OX5	Bol029100	1347	CTTGCTCCAACGCTTTCCTT	CCTCAGCTCTCCAGTGTTCA	280
AOP2	Bol031350	1380	GACACTACACAGAGCCTCGT	CCCCGGGAAGCTTCTCATAT	234
	Bo2g102190		GGAACGTGTCTCCAAAACCC	TAGCACCATCACCAGCATCA	354
GSL-OH	Bo3g052110		CCAGGAAGTGAGAAGTGGGT	ACCAACATCCGCACCAGTAT	552
	Bo9g006240		CCAGGAAGTGAGAAGTGGGT	TAGCACCATCACCAGCATCA	517
	Bol033373	243	GATTGTGCAAAAGGCTTGT	AGAGCATTAGGATTAGGAGGA	188
			Indolic biosynthesis-related genes (17 genes)		
ST5a	Bol026200	1017	GTCCGGTTGCAAGATGGTTT	CCTCTCCGGGTTCTCTTTGT	214
	Bol039395	1014	TGCCGTTTGTGAAGAGGTTG	CCCAATCTCCAACCTTCCCT	210
CYP81F4	Bol032712	1506	CGGTGGAGGAGAAGGAGAAA	CTGACACATGGCTCGTAACG	226
	Bol032714	960	ACCCTGGTGAATACTTGCCA	GAAACACACTGAAGCAGAAC	239
CYP81F1	Bol028918	1503	GTTTGCGGCATCAGAGACAT	GAATAGTCCACGCGTTCACC	299
	Bol017375	369	AAGCAGAGCGGTTCAAGAAG	GCGTGACCATTGTGTTACCA	204
	Bol017376	246	CCGTCTCCTTCAACGGTTCT	CGACGTATTTACCGGTGAGC	170
Title?	Bol028913	1500	GAGACCTCCGCAGTAACCTT	GTCCTCCGTCGGTCTTCTAG	222
	Bol028914	1497	CTTTCCAACTGACGGCCAAA	CGTTAGGTCCGAGAAAAGCG	257
CYP81F2	Bol012237	933	GCAGCCGTGACACTAGAATG	TCCGCCAATCTTGAGGTCTT	231
	Bol014239	1482	TTGTACCGCGTTCTCCTTCT	GACACCATCCTCTGACCCAA	238
CYP81F3	Bol026044	1482	TTCTCCCTACGTTACGGCTC	CTACGAACGGAGAGGAGTCC	251
	Bol028919	1500	TAACAGCGGAGGAGAAGACG	CACCTTCTAACTGGGCCTGA	260
	Bol032711	1492	CCGTCTCACCAACTTCCTCT	CTTCTCAAAGCTCCCTCCCA	292
IGMT1	Bol007029	1119	GTGTTCCTCTCACCTTCCGA	GTGTTGAGGAAGACGCTGTC	260
	Bol020663	342	AGATGCCATGATCTTGAAACGT	CCAGCAATGATAAGCCTGACA	298
IGMT2	Bol007030	1125	AGCCTTTCCCATGGTTCTCA	TCTCTCGCCCTTTCCAAACT	223

Table S7. Physical properties of glucosinolates after mass spectrometry analysis (HPLC/MS, Agilent 1200 series, Agilent Technologies) identified in the leaf samples of *B. oleracea* subspecies by HPLC.

No.	Retention Time (min)	Names	Molecular Weight	Structure
1	8.960	Glucoiberin	344	$[M + H]^+$
2	9.365	Progoitrin	310	$[M + H]^+$
3	9.633	Glucoraphanin	358	$[M + H]^+$
4	10.068	Sinigrin	302	$[M + Na]^+$
5	12.430	Gluconapin	316	$[M + Na]^+$
6	13.385	Glucoiberverin	350	$[M + Na]^+$
7	13.586	4-hydroxyglucobrassicin	385	$[M + H]^+$
8	15.194	Glucoerucin	364	$[M + Na]^+$
9	16.250	Glucobrassicin	369	$[M + H]^+$
10	17.138	4-methoxyglucobrassicin	399	$[M + H]^+$
11	19.399	Neoglucobrassicin	399	$[M + H]^+$

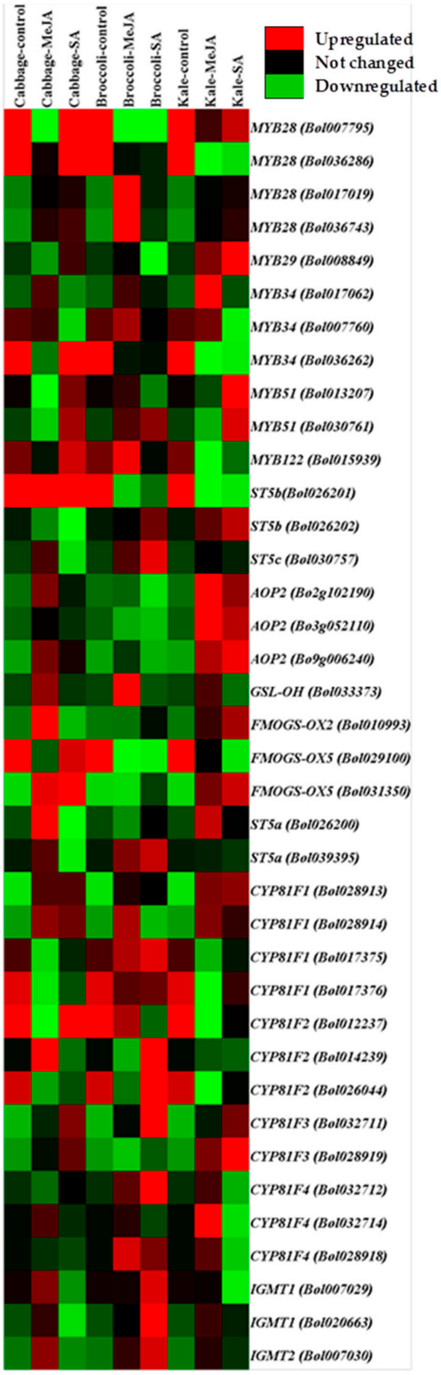

Figure S1. Heat map representation of expression pattern of glucosinolate biosynthesis-related genes under exogenous application of MeJA and SA in cabbage, broccoli and kale. Data were the relative expression of glucosinolate biosynthesis-related genes obtained from qPCR analysis.

Chemical name	Common name	Abbreviations	Chemical structures
3-methylthiopropyl glucosinolate	glucoiberverin	3MTP	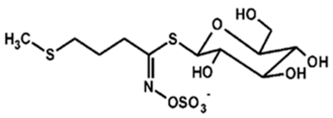
4-methylthiobutyl glucosinolate	glucoerucin	4MTB	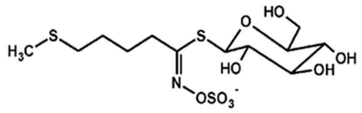
3-(methylsulfinyl)propyl glucosinolate	glucoiberin	3MSOP	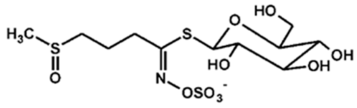
4-(methylsulfinyl)butyl glucosinolate	glucoraphanin	4MSOB	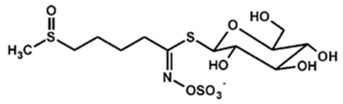
allyl glucosinolate	sinigrin	3PREY	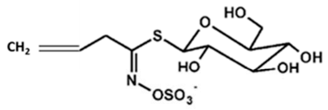
3-butenylglucosinolate	Gluconapin	3BTEY	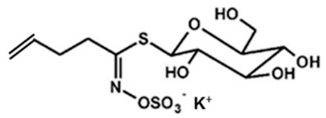
4-hydroxybutyl glucosinolate	progoitrin	4OHB	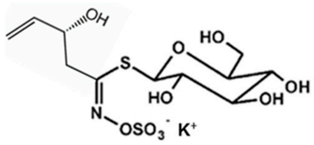

(**a**)

Chemical name	Common name	Abbreviations	Chemical structures
3-Indolylmethyl glucosinolate	glucobrassicin	I3M	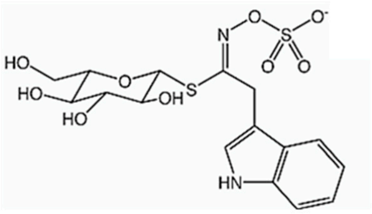
1-methoxy-3-indolylmethyl glucosinolate	neoglucobrassicin	1MOI3M	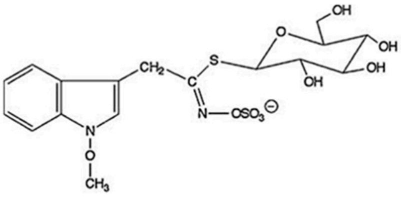
4-methoxy-3-indolylmethyl glucosinolate	4-methoxy glucobrassicin	4MOI3M	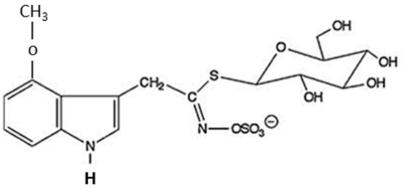

(**b**)

Figure S2. Chemical structures of the glucosinolate compounds and intermediates. (**a**) Aliphatic glucosinolates; (**b**) Indolic glucosinolates.

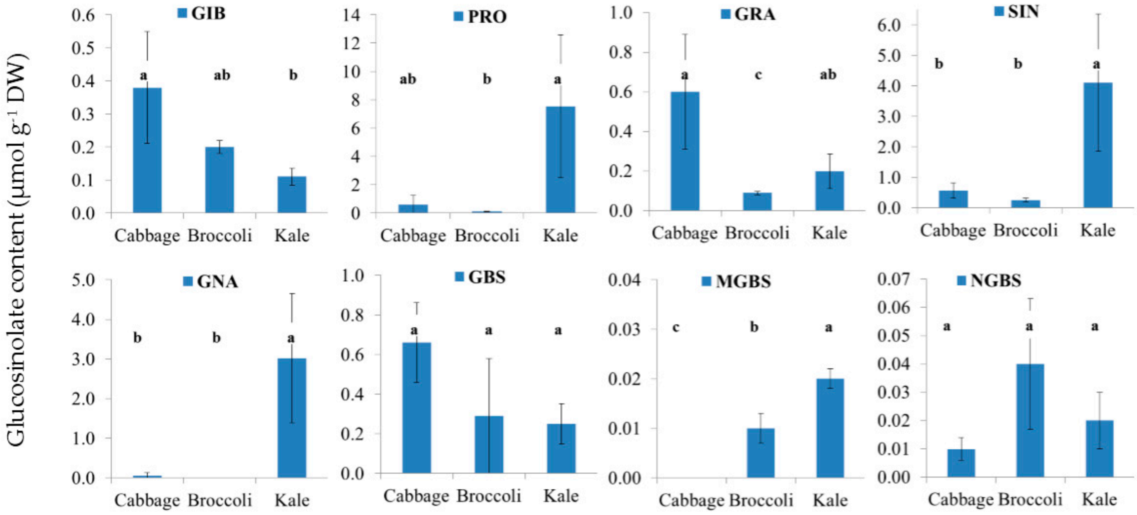

Figure S3. Contents of glucosinolates in control plants cabbage, broccoli and kale.

MYB28 (Bol007795)

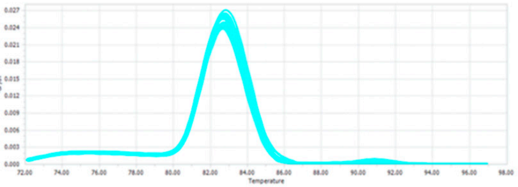

MYB28 (Bol036286)

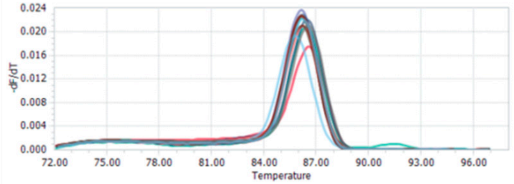

MYB28 (Bol017019)

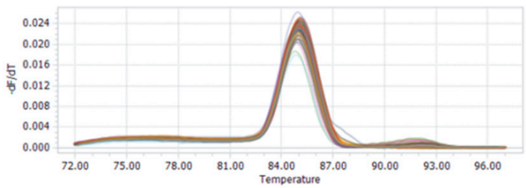

MYB28 (Bol036743)

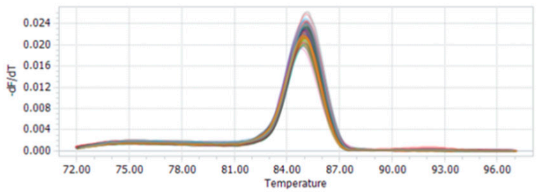

MYB29 (Bol008849)

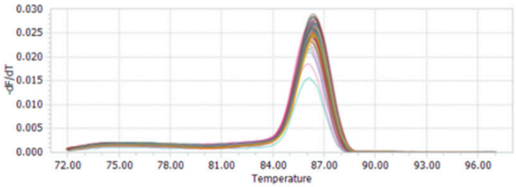

MYB34 (Bol007760)

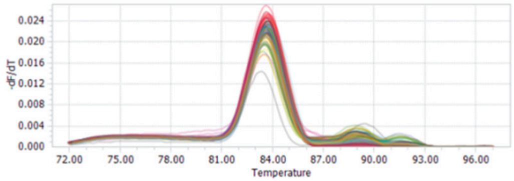

MYB34 (Bol017062)

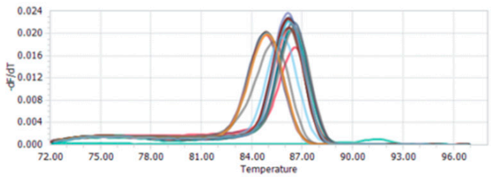

MYB34 (Bol036262)

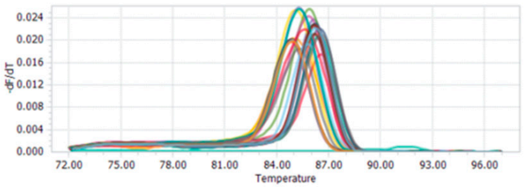

MYB51 (bol013207)

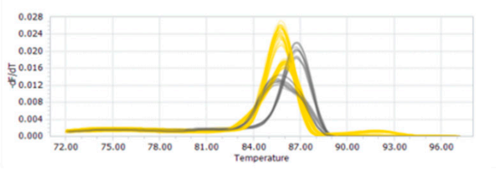

ST5b (Bol026202)

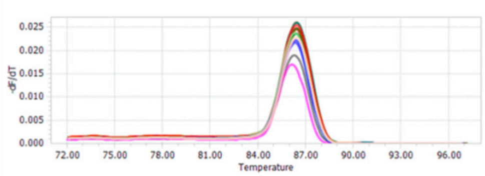

MYB51 (bol030761)

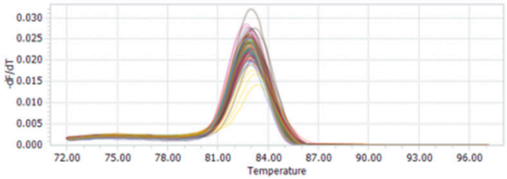

ST5c (Bol030757)

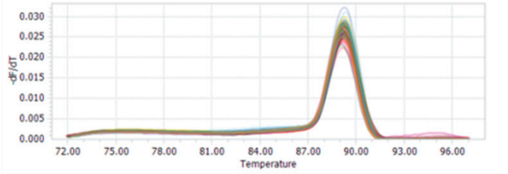

MYB122 (bol026204)

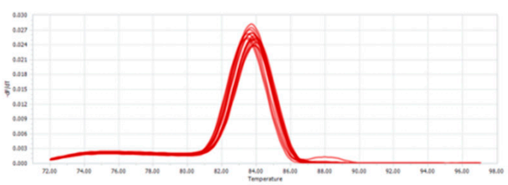

FMOGS-OX2 (Bol010993)

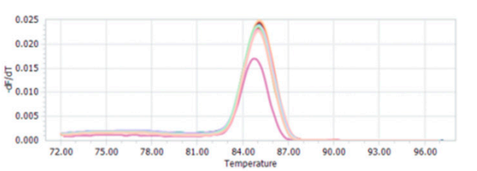

ST5b (bol026201)

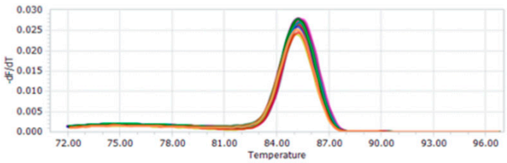

FMOGS-OX5 (bol029100)

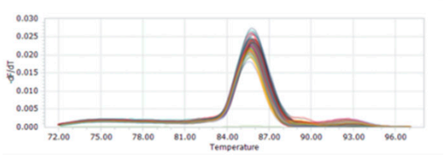

AOP2 (Bo2g102190)

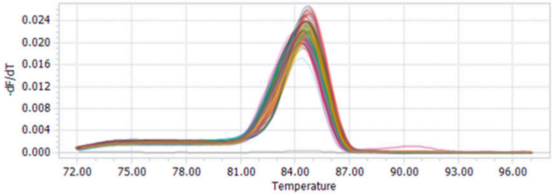

AOP2 (Bo3g052110)

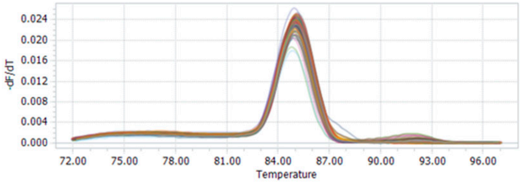

AOP2 (Bo9g006240)

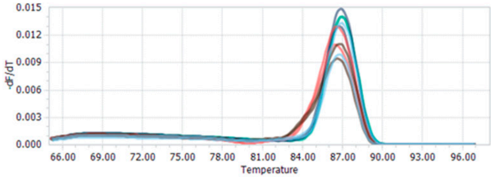

GSL-OH (Bol033373)

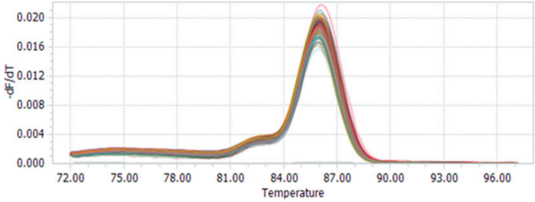

ST5a (Bol026200)

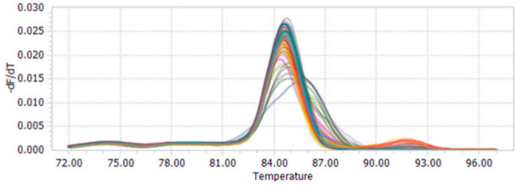

ST5a (Bol039395)

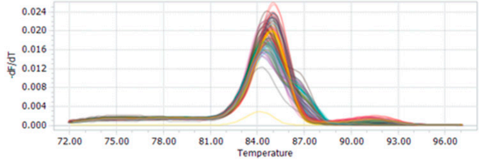

CYP81F4 (Bol032712)

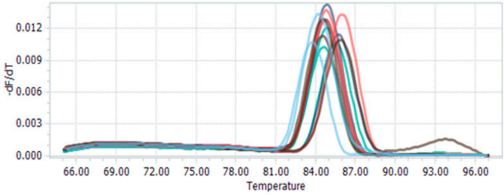

CYP81F4 (Bol032714)

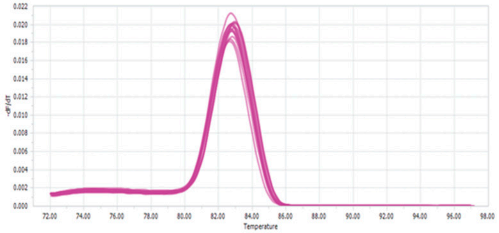

CYP81F4 (Bol028918)

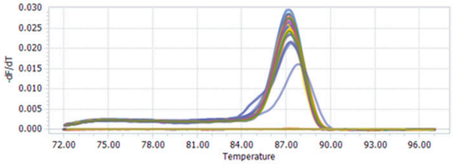

CYP81F1 (Bol017375)

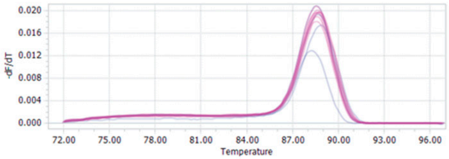

CYP81F1 (Bol017376)

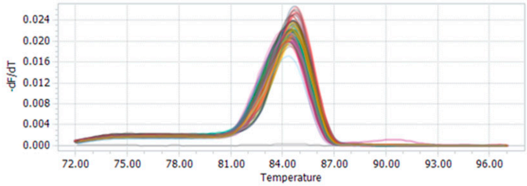

CYP81F1 (Bol028913)

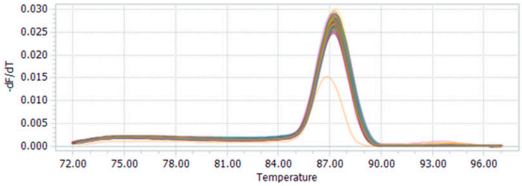

CYP81F1 (Bol028914)

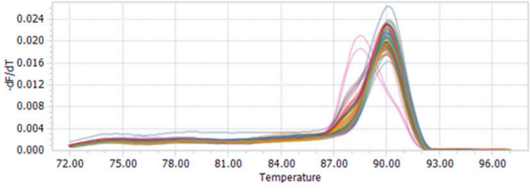

CYP81F2 (Bol012237)

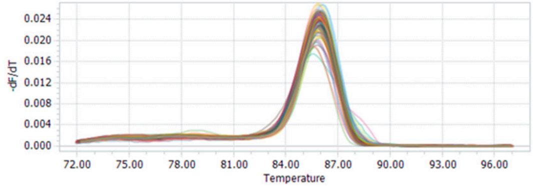

CYP81F2 (Bol014239)

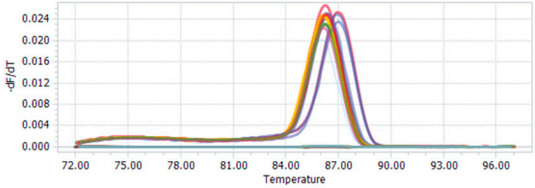

CYP81F2 (Bol026044)

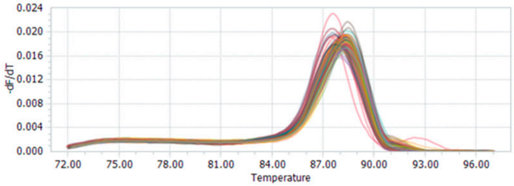

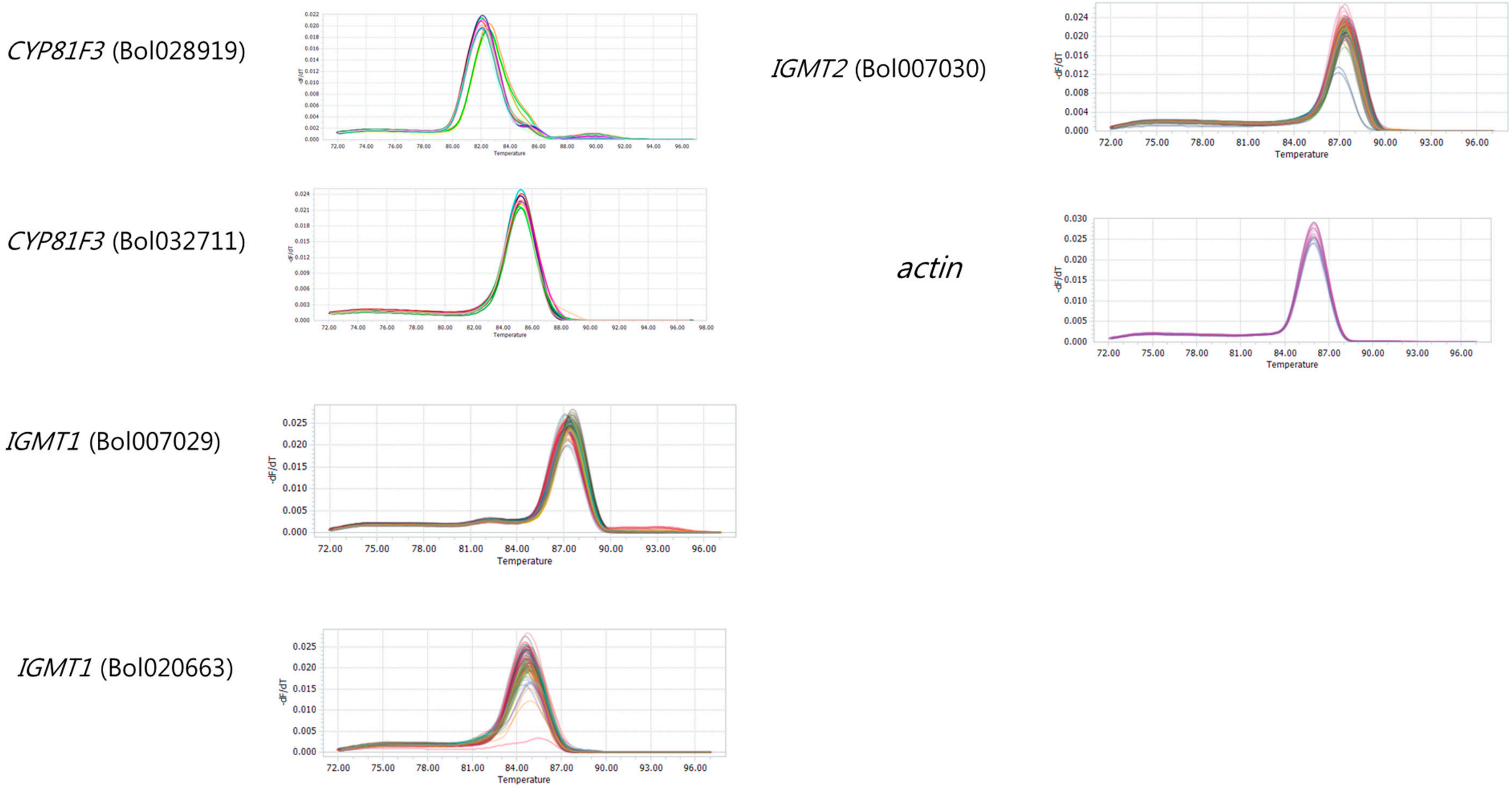

Figure S4. Melting curves obtained for 38 glucosinolate biosynthesis related genes and actin.

Reference

1. Robin, A.H.K.; Yi, G.E.; Laila, R.; Yang, K.; Park, J.I.; Kim, H.R.; Nou, I.S. Expression Profiling of Glucosinolate Biosynthetic Genes in *Brassica oleracea* L. var. capitata Inbred Lines Reveals Their Association with Glucosinolate Content. *Molecules* **2016**, *21*, 787, doi:10.3390/molecules21060787.
